# Effect of the INTER-ACT lifestyle intervention on maternal mental health during the first year after childbirth: A randomized controlled trial

**DOI:** 10.1371/journal.pone.0284770

**Published:** 2023-07-28

**Authors:** Hanne Van Uytsel, Lieveke Ameye, Roland Devlieger, Margriet Bijlholt, Katleen Van der Gucht, Yves Jacquemyn, Annick Bogaerts

**Affiliations:** 1 REALIFE research group, Research Unit Woman and Child, Department of Development and Regeneration, KU Leuven, Leuven, Belgium; 2 Department of Obstetrics and Gynecology, University Hospital Leuven, Leuven, Belgium; 3 Department of Obstetrics and Gynecology, GZA Hospitals Sint- Augustinus, Antwerp, Belgium; 4 Centre for Research and Innovation in Care (CRIC), Faculty of Medicine and Health Sciences, University of Antwerp, Antwerp, Belgium; 5 Faculty of Psychology and Educational Sciences, KU Leuven, Leuven, Belgium; 6 Leuven Mindfulness Centre, KU Leuven, Leuven, Belgium; 7 Department of Obstetrics and Gynecology, University Hospital Antwerp, Antwerp, Belgium; 8 Global Health Institute (GHI), Faculty of Medicine and Health Sciences, University of Antwerp, Antwerp, Belgium; 9 Faculty of Health, University of Plymouth, Devon, United Kingdom; PhD, PLOS, UNITED KINGDOM

## Abstract

We assess whether the INTER-ACT postpartum lifestyle intervention influences symptoms of depression and anxiety, sense of coherence and quality of life during the first year after childbirth. A total of 1047 women of the INTER-ACT RCT were randomized into the intervention (n = 542) or control arm (n = 505). The lifestyle intervention consisted of 4 face-to-face coaching sessions, supported by an e-health app. Anthropometric and mental health data were collected at baseline, end of intervention and 6-months follow-up. We applied mixed models to assess whether the evolution over time of depressive symptoms, anxiety, sense of coherence and quality of life differed between the intervention and control arm, taking into account the women’s pre-pregnancy BMI. There was no statistical evidence for a difference in evolution in anxiety or quality of life between intervention and control arm. But an improvement in symptoms of depression and sense of coherence was observed in women who received the intervention, depending on the mother’s pre-pregnancy BMI. Women with normal/overweight pre-pregnancy BMI, reported a decrease in EPDS between baseline and end of intervention, and the decrease was larger in the intervention arm (control arm: -0.42 (95% CI, -0.76 to -0.08); intervention arm: -0.71 (95% CI, -1.07 to -0.35)). Women with pre-pregnancy obesity showed an increase in EPDS between baseline and end of intervention, but the increase was less pronounced in the intervention arm (control arm: +0.71 (95% CI, -0.12 to 1.54); intervention arm: +0.42 (95% CI -0.42 to 1.25)). Women with a normal or obese pre-pregnancy BMI in the intervention arm showed a decrease in sense of coherence between baseline and end of intervention (-0.36) (95% CI, -1.60 to 0.88), while women with overweight pre-pregnancy showed an increase in sense of coherence (+1.53) (95% CI, -0.08 to 3.15) between baseline and end of intervention. Receiving the INTER-ACT postpartum lifestyle intervention showed improvement in depressive symptoms, in normal weight or overweight women on the short run, as well as improvement in sense of coherence in women with pre-pregnancy overweight only.

**Trial registration:** ClinicalTrials.gov;NCT02989142.

## Introduction

Globally, 20% of women experience mental health problems in the first year after childbirth [1]. Almost half of women who had pre-existing excessive gestational weight gain (GWG) report mental problems 6 weeks after childbirth [2]. Poor mental health during the peripartum period can result in the development of chronic mental disorders in the long run, in both mother and child [3, 4].

Research shows that psychological, biological, social, behavioural, and demographic factors can play a role in the development of maternal mental health problems [5–7]. In addition, there is evidence that a high pre-pregnancy BMI is associated with increased levels of pre-and postnatal depression [8] and that excessive GWG is associated with increased levels of anxiety after childbirth compared to women with adequate GWG [9].

These findings suggest that lifestyle interventions may impact maternal mental health. Many lifestyle interventions have been developed to reduce GWG and subsequently related perinatal complications such as gestational diabetes and pregnancy-induced hypertension [10, 11]. Those who focus on diet and physical activity show a positive effect in the reduction of GWG but there are limited effects on the reduction of perinatal complications [12–14]. One could argue that prenatal lifestyle interventions start too late [15] because behaviour changes take time. Another limitation in existing lifestyle interventions is the lack of attention to women’s mental health [16, 17].

Depressive and/or anxiety symptoms are associated with unhealthy eating patterns [18, 19], which can have an impact on weight and in turn lead to even higher levels of depression and anxiety [8, 9]. To interrupt this vicious circle, mental health problems should be an integral target of lifestyle interventions, in addition to diet and physical activity.

Unfortunately, mental health outcomes such as depression and anxiety are hardly evaluated in lifestyle interventions [12, 14, 17, 20]. To our knowledge none of the studies have investigated the effect of lifestyle interventions on SOC and very few investigated the impact on the quality of life (QoL) [21].

INTER-ACT is a randomized controlled trial (RCT) [10] in which an eHealth supported lifestyle intervention, with focus on diet, exercise and mental health is starting in the early postpartum period (6 weeks-6 months after childbirth) and initiated again in the next pregnancy (1^st^, 2^nd^ & 3^rd^ trimester) in women with a history of excessive GWG. The primary aim of INTER-ACT was to reduce pregnancy- and birth related complications at the end of a subsequent pregnancy. This study aims to assess whether the INTER-ACT lifestyle intervention affects maternal mental health outcomes in the form of depressive symptoms, anxiety, SOC and QoL during the first year after childbirth. Our hypothesis is that women who received the INTER-ACT postpartum lifestyle intervention have better mental health outcomes during the first year after childbirth compared to their counterparts who received no intervention.

## Materials & methods

### Study design and setting

The INTER-ACT study (ClinicalTrials.gov (NCT02989142)) is a multicentre RCT to investigate lifestyle and lifestyle interventions after childbirth and during a subsequent pregnancy to reduce pregnancy- and birth related complications. Details of the INTER-ACT methodology are published elsewhere [10]. For current analyses we focused on the first year after childbirth to assess the impact of the INTER-ACT postpartum lifestyle intervention on the mental health outcomes at the end of intervention and after 6 months of follow-up. Measurements were performed at baseline (6 weeks after childbirth), at the end of intervention (6 months after childbirth) and at 6 months follow-up (12 months after childbirth). Women were recruited between May 2017 and April 2019 in 6 hospitals in Belgium: University Hospital Leuven, University Hospital Antwerp, Gasthuiszusters Hospital Antwerp, St-Franciscus Hospital Heusden-Zolder, Jessa Hospital Hasselt and Hospital Oost- Limburg Genk. The study was conducted according to the guidelines of the Declaration of Helsinki and was approved by the clinical trial center/ ethical committee UZ Leuven (protocol code B322201730956/ S59889). All participants gave written consent.

### Participants

**Fig 1:**
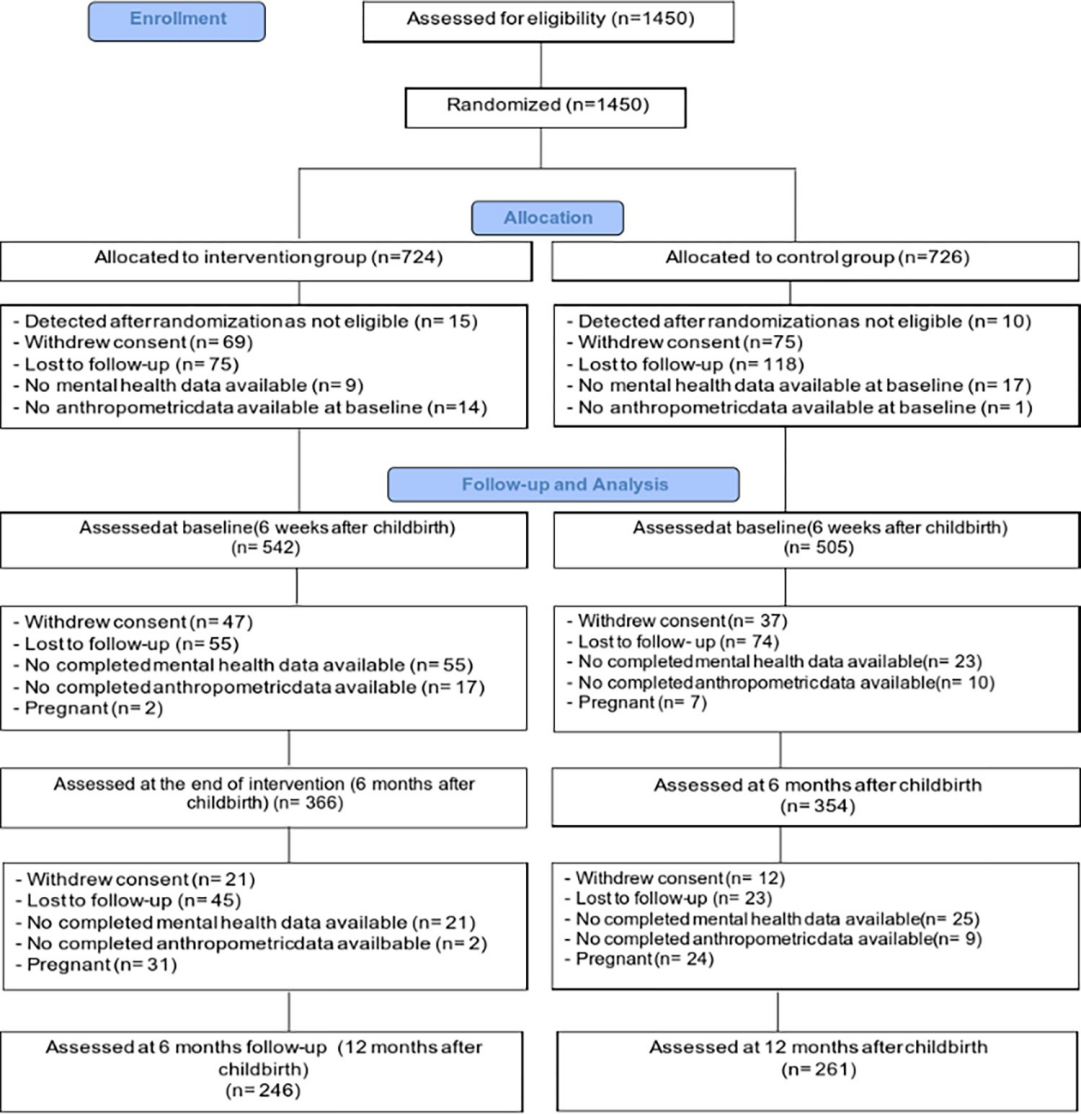
Flow chart of participant follow-up.

Study nurses informed and recruited participants 2 to 3 days after childbirth. Inclusion criteria were: excessive GWG according to the 2009 National Academy of Medicine (NAM) guideline [22], age ≥18 years and proficiency in Dutch. Exclusion criteria were twin birth, stillbirth, pre-pregnancy underweight (BMI ≤18.5), (planned) bariatric surgery, or the presence of any of the following serious conditions: diabetes mellitus, kidney disease, mental illness. A total of 1450 women were included.By using computer- generated randomization (details in 2.3), participants were assigned to the intervention arm (n = 724) or control arm (n = 726). After randomization 25 participants were considered as not eligible and therefore excluded. Reasons for exclusion were pre-pregnancy underweight (intervention arm: n = 9; control arm: n = 7), no pre-existing excessive GWG (intervention arm: n = 1; control arm n = 3), chronic disease (intervention arm: n = 1), not familiar with the Dutch language (intervention arm: n = 2), incorrect timing of measurement (intervention arm: n = 1) and informed consent not completed (intervention arm: n = 1). As a result, 1047 women remained for the present analyses as they received the baseline measurement at 6 weeks after childbirth (baseline) and completed the mental health questionnaires. Subsequently, at the end of intervention data were collected from 720 participants (i.e., mental health questionnaires and anthropometrics), followed by 507 participants at 6 months follow-up (mental health questionnaires and anthropometrics) (Fig 1).

### Randomization

Study nurses were midwives employed in the concerned hospital department who volunteered to collaborate in the INTER-ACT study. They were trained by research staff in recruiting and including participants, as well as collecting and reporting data from the medical records. At time of recruitment and inclusion, both participants and study nurses were blinded for randomization. Study nurses entered the data of the recruited participants into the electronic Case Report Form (eCRF) ‘Castor’ (https://data.castoredc.com/). Randomization into the intervention or control arm was electronically performed by the biostatistician within the first week after childbirth, using an allocation ratio of 1:1 supported by block randomization (block size 4, 6 and 8) and stratification by hospital. The study personnel announced the randomization directly to the participants during the first study visit (6 weeks after childbirth). Blinding was not possible for participants, research staff and caregivers due the difference in follow-up between the intervention arm (questionnaires, body measurement, face-to-face coaching sessions, app with Bluetooth connected weight scale and pedometer) and control arm (only questionnaires and body measurement).

### Intervention

The intervention consisted of 4 face-to-face coaching sessions which focussed on nutrition, physical activity and mental health, in combination with an e-health app. Study visits were conducted by researchers, midwives, and nurses who received a three-day intensive training course. To standardize the coaching sessions, all trained staff received a detailed manual including the steps, techniques and content to discuss in each session.

More in detail: session 1 and session 2 (6 and 8 weeks after childbirth) focused on a general discussion of the lifestyle components: nutrition, physical activity and mental health. Session 3 (12 weeks after childbirth) offered a specific targeted and in depth coaching on nutrition, eating behaviour and food intake. Session 4 (6 months after childbirth) offered a specific targeted coaching on physical activity, sedentary behaviour and mental health.

The mental health component consisted of 2 main topics; stressors and social support. The session (4) aimed to provide insight into specific stressors that have an impact on the participant’s well-being and to increase knowledge around the impact of well-being on general health. We informed participants about feelings of stress and tension related to transition to parenthood and identified and discussed personal stressors.

In addition, the session focused on strengthening the participant’s social network by identifying and optimizing the social support they received. Information was provided using the Ask- Tell- Ask method.

Motivational interviewing, behavioural change techniques, goal settings, action planning and reinforcement were implemented in the coaching sessions. Additionally, the e-health app supported the coaching sessions by sending motivational messages and informational advice, and participants were able to set and monitor their own lifestyle goals. To stimulate self-monitoring of physical activity and body weight, women were supported to use a received Bluetooth connected weight scale (Withings Body+, Withings, Issy-les-Moulineaux, France) and activity tracker (Withings GO) which was linked to the e-health app [23].

To increase feasibility for both staff and participants, a deviation of 2 weeks before and 4 weeks after the predetermined visit timepoint was allowed.

### Data-collection

Pregnancy- and birth related outcomes were extracted from medical records by trained study nurses at the time of recruitment.

Body mass index (BMI) was calculated as weight (kg)/ height (m^2^). Pre-pregnancy weight was self-reported and weight at childbirth was self-reported or measured by medical personnel on admission at the birth unit. Based on these two weight measurements, total weight gain was calculated and assessed whether women had excessive GWG according to the NAM guidelines [22].

The socioeconomic status (age, education level, ethnicity, employment status, family composition and family income) was surveyed once at baseline.

The methodology of the mental health questionnaires was also described in previous research [24]. Characteristics of the mental health questionnaires are presented in Table 1.

**Table 1:** Characteristics of the mental health questionnaires.

	Depression		Anxiety		SOC	QoL
	EPDS	GMDS	STAI-6	EDS-3A	SOC-13	QoL
Items	10	13	6	3	13	1
**Scale**	4- point Likert Scale	4- point Likert Scale	4- point Likert Scale	4- point Likert Scale	7- point Likert Scale	Lineair Analogue Scale
**Range numeric score**	0–30 ^a^	0–39 ^a^	20–80 ^a^	0–9 ^a^	13–91^b^	0–100 ^a^
**Cut-off score**	≥ 10	< 13 13–26 ≥ 27	≥40	≥5	< 70	Median ^c^

EPDS = Edinburgh Postnatal Depression Scale; GMDS = Gotland Male Depression Scale; sSTAI = spielberger State-Trait Anxiety Inventory; EDS-3A = Edinburg Depression Scale- 3 Anxiety subscale; SOC = Sense Of Coherence; QoL = Quality of Life

a: The higher the score, the higher the level of depression/ anxiety/ QoL

b: The higher the score, the better the sense of coherence

c: Cut-off is the median calculated at each individual timepoint: baseline (6 weeks after childbirth), end of intervention (6 months after childbirth) and 6 months follow-up (12 months after childbirth).

As shown in previous research, the Edinburgh Postnatal Depression Scale (EPDS) is appropriate for measuring levels of depression and anxiety after childbirth [25]. A cut-off score of ≥ 10 is recommended for the detection of mild to severe PPD [26]. The questionnaire contained one question about self-harming, generating an integrated alert in the eCRF system ‘Castor’ initiating immediate response from the research staff.

The Gotland Male Depression Scale (GMDS) complements the EPDS as it is a valid instrument for measuring non-typical suicidality-related symptoms of depression and using both scales can increase the detection rate of depressive symptoms [27]. Cut-off scores were defined in 3 classes: <13 = no signs of depression; 13–26 indicates possible major depression; ≥27 clearly indicates depression [27].

The Spielberger State Trait Anxiety Inventory 6-item (sSTAI-6) [28] is a validated and reliable measurement for maternal anxiety after childbirth [29]. We used the shortened 6-item version of the sSTAI to make it less cumbersome for participants. A cut-off score of 40 is commonly used to predict postnatal anxiety and mood disorder with the original 20-item questionnaire [29, 30]. Therefore, the numeric sum score of the 6-items was converted to a range between 20 to 80 by using the following formula [31]: 6-item sum score / 6 x 20. Previous research indicates that three questions from the EPDS questionnaire are also sensitive for measurement of anxiety [32]. The three items (3-4-5) are brought together under the EDS-3A and were used in the current analysis to enhance validity. We used a cut-off score of 5, due the specificity of 90% and less misclassification than a lower cut-off as shown in previous research [32].

The Sense Of Coherence (SOC) is measured with the 13 item short scale (SOC-13), derived from the original 29-items Orientation to Life Questionnaire [33]. For current analysis the format of the Institute for Data Collection and Research (centERdata) was used [34]. A SOC score <70 indicates that participants are less able to understand, influence and make sense of situations, while a high sense of coherence (≥70) indicates a better ability to cope with stressful situations in life.

Quality of Life (QoL) was assessed using a Linear Analogue Scale (LAS). Participants were asked to score QoL on a scale from 0 to 100, with 0 representing a poor quality of life and 100 an extremely good quality of life. The LAS was one comprehensive score, summarizing physical, psychological, and social aspects.

All questionnaires used in this study were self-completed questionnaires in Dutch version. Each participant received by e-mail a personal link to the questionnaire, which was sent from the eCRF. In current analyses, the Cronbach’s alpha was acceptable (α = ≥.70 < .80) for the EDS-3A, to good (α = ≥.80 < .90) for the EPDS, GMDS, sSTAI-6 and SOC at each timepoint.

### Data-analysis

Data were analysed by using Statistical Package for the Social Science (SPSS) version 27.0 (IBM, Armonk, New York) and Statistical Analysis System (SAS) version 9.4 (Cary, New York).

Differences in mental health outcomes between the intervention and the control arm were investigated for all pre-pregnancy BMI groups together, as well as stratified by pre-pregnancy BMI class: normal pre-pregnancy weight (BMI 18.5–24.9 kg/m^2^), pre-pregnancy overweight (BMI 25.0–29.9 kg/m^2^) and pre-pregnancy obesity (BMI ≥30 kg/m^2^). Differences in categorical variables were assessed with the (Mantel-Haenszel) chi-square test; differences in continuous variables were assessed with the t-test or Mann-Whitney U test.

To assess whether the evolution in mental health outcomes during the first year after childbirth differs between the intervention arm and the control arm, we applied mixed models. For each mental health outcome variable, we constructed a mixed model considering as explanatory variables: time since birth (in months), the randomization arm (intervention vs. control), the pre-pregnancy BMI class (normal, overweight or obese) and baseline characteristics that showed imbalance between the two randomization arms (mode of conception, the amount of excessive weight at the end of pregnancy (above the IOM cutoff for excessive GWG (in kg)), and QoL at W6 (Week 6)). We also considered a breakpoint at month 6 (in order to allow for a difference in the modeled evolution in the first 6 months after childbirth vs M6 until M12, and inter-action terms between time, randomization arm and pre-pregnancy BMI class. We started from the full model and applied backward variable selection by dropping one per one the term with the highest associated P-value. In case an interaction term showed statistical significance, the main effect terms were not dropped from the model. We used an unstructured covariance structure in order to take into account the dependency between subsequent measurements of the same mother.

A p-value of < 0.05 was considered statistically significant. No adjustment for multiple testing was performed.

## Results

### Participant characteristics

Baseline characteristics of the participants are shown in Table 2.

**Table 2:** Participant characteristics and the differences between the intervention- and control arm.

		Overall		Intervention group		Control group	
		(n = 1047)		(n = 542)		(n = 505)	
		**mean**	**SD**	**mean**	**SD**	**mean**	**SD**
**Age at birth, mean (SD)**		31.3	3.9	31.2	4	31.4	3.9
		**n**	**%**	**n**	**%**	**n**	**%**
**Parity**	Primiparous	572	54.6	290	53.5	282	55.8
	Multiparous	475	45.4	252	46.5	223	44.2
**Education**	Secondary level	300	28.7	153	28.2	147	29.1
	Bachelor’s level	407	38.9	209	38.6	198	39.2
	Master’s level and above	340	32.5	180	33.2	160	31.7
**Employment status**	Employed	964	92.1	502	92.6	462	91.5
	Unemployed	83	7.9	40	7.4	43	8.5
**Etnicity**	White European	963	92	493	9	470	93.1
	Other etnicity	84	8	49	91	35	6.9
Method of conception *	Spontaneous	920	90.2	464	88.4	456	92.1
	ART	100	9.8	61	11.6	39	7.9
* *	*Missing*	*27*		*17*		*10*	
**Method of delivery**	Spontaneous	702	67	356	65.7	346	68.5
	Vacuum- extraction/ forceps	99	9.5	54	10	45	8.9
	Primairy section (planned)	119	11.4	61	11.3	58	11.5
	Secondary section (urgent)	127	12.1	71	13	56	11.1
**Exclusive breastfeeding**	Yes	561	53,6	279	51.5	282	55.8
**at 6 weeks postpartum**	No	486	46,4	263	48.5	223	44.2
**Family composition**	Biological parents						
** **	Single parent	75	7.2	46	8.5	29	5.7
	family						
	Two parent	893	85.3	456	84.1	437	86.5
	family						
	Blended family	79	7.5	40	7.4	39	7.7
**Family income (monthly)**	<2000 euro	69	7	35	6.9	34	7.1
	2000–2999 euro	179	18.2	89	17.5	90	18.8
	3000–3999 euro	454	46	239	47	215	45
	4000 or above	284	28.8	145	28.5	139	29.1
	*Missing*	*61*		*34*		*27*	
**History of depressive feelings**	Yes	153	15.3	78	15	75	15.5
	No	850	84.7	442	85	408	84.5
* *	*Missing*	*44*		*22*		*22*	
**History of anxiety feelings**	Yes	112	11.2	59	10.9	53	11
	No	888	88.8	459	84.7	429	89
* *	*Missing*	*47*		*24*		*23*	
**Pre- pregnancy BMI**	NW	506	48.3	259	47.8	247	48.9
	OW	375	35.8	193	35.6	182	36
	OB	166	15.9	90	16.6	76	15
		**median**	**IQR**	**median**	**IQR**	**median**	**IQR**
**Gestational weight gain in kg** *	Among NW	18.5	17–20.9	18	17–20	19	17–21
	Among OW	16	13.5–19	16	14–19	16	13–19
	Among OB	14	17–21	14	11–17	14	12–17
**Depression score at baseline**	EPDS (0–30)	6	4–10	7	4–10	6	4–10
	GMDS (0–39)	5	3–9	5	3–8	5	2–9
**Anxiety score at baseline**	sSTAI (20–80)	37	30–43	37	33–47	37	30–47
	EDS-3A (0–9)	4	2–5	4	2–5	4	2–5
**Quality of life score at baseline** *	(0–100)	80	70–85	78	70–84	80	71–85
**Sense of Cohrence score at baseline**	SOC (13–91)	70	61–77	70	61–77	70	63–78

* Statistically significant difference between control and intervention group: method of conception P = 0.046; gestational weight gain in kg P = 0.035; QoL at baseline P = 0.038

The drop-out rate at the end of intervention was higher in women with pre-pregnancy overweight (32.9%) or obesity (41.6%) compared to women with a normal pre-pregnancy BMI (26.6%) (P = ≤0.001).

Despite randomization, there seems to be an imbalance between the intervention- and control arm for method of conception, gestational weight gain in kg and QoL at baseline. More women were more spontaneously pregnant in the control arm (92.1%) compared to the intervention arm (88.4%; P = 0.046). In women with a normal pre-pregnancy BMI, the median GWG was 18kg (IQR 17–20) in the intervention arm compared to 19kg (IQR 17–21) in the control arm(P = 0.035). The median score of QoL at baseline was slightly higher in the control arm (80; IQR 71–85) compared with the intervention arm (78; IQR 70–84) (P = 0.038). No difference was found anymore between intervention- and control arm regarding method of conception, quality of life and GWG in women with a 6 month measurement (n = 720) or 12 month measurement (n = 507). Details are presented in Table 2.

### Differences in the prevalence of mental health

In the overall group, no significant differences in the prevalence of anxiety, depressive symptoms, SOC or QoL, were shown between the intervention- and control arm, neither at the end of intervention nor after 6 months follow-up.

However, in the subgroup of women with a normal pre-pregnancy BMI, depressive symptoms (EPDS ≥10) after 6 months follow-up were statistically significantly less prevalent in the intervention arm compared to the control arm (16.7% vs 29.1%, P = 0.016). This difference was not shown in women with pre-pregnancy overweight or pre-pregnancy obesity.

### Differences in numeric mental health scores

In the overall group, no significant differences in numeric mental health scores (EPDS, GMDS, sSTAI-6, EDS-3A, SOC-13, QoL) were shown at the end of intervention or after 6 months follow-up between the intervention- and control arm.

Women with a normal pre-pregnancy BMI who received the INTER-ACT intervention scored one point lower on the GMDS after 6 months follow-up compared to women with normal pre-pregnancy BMI and who received usual care (5 points versus 6 points, P = 0.05). Results are presented in Table 3.

**Table 3:** Univariate analyses of the effect of the INTER-ACT lifestyle intervention on mental health.

			Intervention group		Control group		
Mental health outcomes	Time point	BMI category pre-pregnancy	n	median (IQR)	n	median (IQR)	P- value
		Overall	542	37 (33–47)	505	37 (30–47)	** **
		NW	259	37 (33–47)	247	37 (30–47)	
		OW	193	37 (33–47)	182	37 (30–47)	
		OB	90	40 (33–51)	76	37 (33–43)	
	End of intervention *(6 months after childbirth)*	Overall	366	40 (33–50)	354	40 (33–47)	0.217
		NW	184	40 (33–47)	188	40 (33–47)	0.978
		OW	134	40 (33–50)	117	37 (32–47)	0.147
		OB	48	43 (33–57)	49	40 (33–48)	0.238
	6 months follow-up *(12 months after childbirth)*	Overall	246	40 (33–50)	261	40 (33–47)	0.576
		NW	126	40 (33–47)	141	40 (33–50)	0.291
		OW	90	40 (33–51)	86	37 (30–47)	0.179
		OB	30	42 (33–55)	34	37 (33–50)	0.207
**EDS-3A**	Baseline *(6 weeks after childbirth)*	Overall	542	4 (2–5)	504	4 (2–5)	
		NW	259	4 (2–5)	247	3 (2–5)	
		OW	193	3 (2–5)	182	4 (2–5)	
		OB	90	4 (3–6)	75	4 (2–5)	
	End of intervention *(6 months after childbirth)*	Overall	366	3 (2–5)	354	3 (2–5)	0.720
		NW	184	3 (2–5)	188	3 (1–5)	0.528
		OW	134	3 (1–5)	117	3 (2–5)	0.628
		OB	48	4 (2–6)	49	3 (2–6)	0.535
	6 months follow-up *(12 months after childbirth)*	Overall	246	3 (2–5)	261	3 (2–5)	0.64
		NW	126	3 (2–4)	141	3 (2–5)	0.748
		OW	90	3 (2–5)	86	3 (2–4)	0.514
		OB	30	4 (2–6)	34	3 (2–5)	0.400
**EPDS**	Baseline *(6 weeks after childbirth)*	Overall	542	7 (4–10)	504	6 (4–10)	
		NW	259	6 (4–10)	247	6 (4–10)	
		OW	193	6 (4–10)	182	6 (3–10)	
		OB	90	9 (5–13)	75	7 (4–11)	
	End of intervention *(6 months follow-up)*	Overall	366	6 (3–10)	354	6 (3–9)	0.848
		NW	184	6 (3–9)	188	6 (3–9)	0.702
		OW	134	5 (3–9)	117	5 (3–10)	0.716
		OB	48	9 (5–12)	49	8 (4–11)	0.623
	6 months follow-up *(12 months after childbirth)*	Overall	246	6 (3–9)	261	6 (3–10)	0.873
		NW	126	6 (3–8)	141	6 (3–10)	0.182
		OW	90	6 (3–10)	86	6 (2–8)	0.391
		OB	30	7 (4–14)	34	6 (2–11)	0.369
**GMDS**	Baseline *(6 weeks after childbirth)*	Overall	256	5 (3–8)	237	5 (2–9)	
		NW	116	4 (2–7)	143	6 (2–9)	
		OW	99	5 (3–9)	92	5 (3–9)	
		OB	41	8 (3–12)	36	7 (3–9)	
	End of intervention *(6 months after childbirth)*	Overall	261	5 (2–9)	237	5 (2–9)	0.679
		NW	132	5 (2–10)	124	5 (3–9)	0.877
		OW	97	5 (2–7)	79	5 (2–9)	0.646
		OB	32	8 (2–14)	34	7 (2–12)	0.933
	6 moths follow-up *(12 months after childbirth)*	Overall	242	5 (2–9)	251	5 (2–10)	0.154
		NW	123	5 (2–8)	137	6 (3–11)	0.050
		OW	90	5 (2–9)	83	5 (2–9)	0.783
		OB	29	5 (2–14)	31	6 (3–11)	0.812
**SOC**	Baseline *(6 weeks after childbirth)*	Overall	542	70 (61–77)	503	70 (63–78)	
		NW	259	72 (63–79)	247	69 (62–76)	
		OW	193	71 (61–77)	181	72 (63–78)	
		OB	90	66 (55–72)	75	70 (60–76)	
	End of intervention *(6 months after childbirth)*	Overall	366	73 (63–79)	353	71 (62–78)	0.138
		NW	184	74 (66–79)	188	72 (63–78)	0.143
		OW	134	73 (62–80)	116	70 (60–79)	0.168
		OB	48	66 (56–73)	49	67 (60–77)	0.242
	6 months follow-up *(12 months after childbirth)*	Overall	246	71 (62–79)	261	72 (61–79)	0.848
		NW	126	72 (64–78)	141	71 (60–79)	0.363
		OW	90	72 (58–80)	86	73 (64–80)	0.522
		OB	30	68 (58–78)	34	71 (60–79)	0.267
**QoL**	Baseline *(6 weeks after childbirth)*	Overall	542	78 (70–84)	502	80 (71–85)	
		NW	259	80 (70–85)	247	80 (72–86)	
		OW	193	79 (70–85)	180	80 (70–85)	
		OB	90	75 (65–81)	75	80 (70–84)	
	End of intervention *(6 months after childbirth)*	Overall	364	79 (70–85)	352	78 (70–85)	0.659
		NW	183	80 (70–85)	187	80 (71–86)	0.941
		OW	134	79 (70–84)	116	77 (70–85)	0.425
		OB	47	74 (60–83)	49	75 (69–83)	0.697
	6 months follow-up *(12 months after childbirth)*	Overall	244	80 (70–84.7)	260	77 (70–85)	0.681
		NW	125	80 (70–85)	140	76 (66–85)	0.450
		OW	90	80 (60–84)	86	78 (70–82)	0.754
		OB	29	75 (31–84)	34	77 (65–77)	0.263

BMI = body mass index; NW = normal weight; OW = overweight; OB = obesity; IQR = interquatile range; EPDS = Edinburgh Postnatal Depression Scale; GMDS = Gotland Male Depression Scale; sSTAI = spielberger State-Trait Anxiety Inventory; EDS-3A = Edinburg Depression Scale- 3 Anxiety subscale; SOC = Sense Of Coherence. Significance level was calculated using the Mann- Whitney U test.

### Effect of the INTER-ACT lifestyle intervention on the evolution of mental health during the first year after childbirth (mixed models)

We assessed the evolution over time of the mental health outcomes with mixed models, considering as explanatory variables the randomization arm (intervention vs control), the pre-pregnancy BMI and baseline characteristics that showed imbalance between the two randomization arms (mode of conception, the amount of excessive weight at the end of pregnancy (above the IOM cutoff for excessive GWG (in kg)), and QoL at W6 (Week 6) (Table 4, S1 File).

**Table 4:** Mixed models of the effect of the INTER-ACT intervention on the evolution of mental health over time.

**Anxiety**									
**Outcome variable**		**Explanatory variables**	**ß**		**SE**		***t***	**p-value**	
sSTAI-6		Intercept	64.873		17.622		36.81	< .0001	
		Months	0.437		0.094		4.67	< .0001	
		W6QoL	-0.356		0.022		-16.42	< .0001	
		Exc	0.164		0.068		2.43	0.02	
		M6*monthMinus6	-0.312		0.147		-2.12	0.03	
EDS-3A		Intercept	7.026		0.354		19.87	< .0001	
		Months	-0.067		0.018		-3.75	0.0002	
		Obese	0.247		0.218		1.13	0.26	
		W6QoL	-0.044		0.004		-9.91	< .0001	
		Months*obese	0.099		0.048		2.07	0.04	
		M6*monthsMinus6	0.088		0.027		3.26	0.001	
		Obese*M6*monthsMinus6	-0.190		0.073		-2.60	0.001	
**Depression**									
**Outcome variable**		**Explanatory variables**	**ß**		**SE**		***t***	**p-value**	
EPDS		Intercept	16.979		0.757		22.43	< .0001	
		Months	-0.093		0.039		-2.40	0.017	
		Intervention	0.267		0.265		1.01	0.314	
		Obese	0.399		0.438		0.91	0.363	
		W6QoL	-0.130		0.009		-14.01	< .0001	
		Months*intervention	-0.066		0.031		-2.10	0.036	
		Months*obese	0.251		0.010		2.51	0.012	
		M6*monthsMinus6	0.176		0.056		3.15	0.002	
		Obese*M6*monthsMinus6	-0.392		0.153		-2.56	0.011	
GMDS		Intercept	19.981		0.996		20.05	< .0001	
		Obese	12.080		0.424		2.85	0.005	
		W6QoL	-0.180		0.013		-14.16	< .0001	
**Sense Of Coherence**									
**Outcome variable**		**Explanatory variables**	**ß**		**SE**	***t***		**p-value**	
SOC		Intercept	34.290		2.070	16.57		< .0001	
		Months	0.218		0.126	1.74		0.083	
		Intervention	3.072		1.009	3.04		0.002	
		Overweight	3.207		1.125	2.85		0.004	
		Obese	0.224		1.291	0.17		0.86	
		W6QoL	0.441		0.024	18.15		< .0001	
		Exc	-0.161		0.078	-2.07		0.04	
		Intervention*overweight	-5.509		1.579	-3.49		0.0005	
		Intervention*obese	-5.096		1.750	-2.91		0.004	
		Months*intervention	-0.299		0.188	-1.59		0.11	
		Months*overweight	-0.611		0.216	-2.83		0.005	
		Months*intervention*overweight	1.032		0.316	3.27		0.001	
		M6*monthsMinus6	-0.210		0.197	-1.06		0.29	
		Intervention*M6*monthsMinus6	0.258		0.286	0.90		0.37	
		Overweight*M6*monthsMinus6	0.697		0.343	2.03		0.04	
		Intervention*obese*M6*monthsMinus6	-1.076		0.485	-2,22		0.03	
**Quality Of Life**									
**Outcome variable**	**Explanatory variables**		**ß**	**SE**		***t***			**p-value**
QoL	Intercept		10.565	0.702		15.06			< .0001
	Months		0.991	0.129		7.70			< .0001
	Overweight		1.476	0.481		3.07			0.002
	Obese		2.040	0.658		3.10			0.002
	W6QoL		0.842	0.008		108.52			< .0001
	Months*overweight		-0.894	0.205		-4.36			< .0001
	Months*obese		-1.438	0.284		-5.06			< .0001
	M6*monthsMinus6		-1.549	0.195		-7.95			< .0001
	Overweight*M6*monthsMinus6		1.398	0.312		4.47			< .0001
	Obese*M6*monthsMinus6		2.505	0.433		5.79			< .0001

Months = months since childbirth; W6QoL = Quality of Life at week 6; Exc = Amount of excessive gestational weight gain above the IOM cutoff, depending on the pre-pregnancy BMI (above 16kg for normal pre-pregnancy BMI, above 11.5 kg for overweight, and above 9kg for obese pre-pregnancy BMI); M6 = 1 if month >6, 0 if months <6, in order to model a breakpoint at month 6; MonthsMinus6 = months– 6; Obese = 1 if pre-pregnancy BMI> = 30, 0 otherwise; Intervention = 1 if intervention arm, 0 if control arm Overweight = 1 if pre-pregnancy BMI between 25 and 29.9, 0 otherwise.

sSTAI-6 = Spielberger State-Trait Anxiety Inventory- 6 item; EDS-3A = Edinburgh Depression Scale-3 Anxiety subscale; EPDS = Edinburgh Postnatal Depression Scale; GMDS = Gotland Male Depression Scale; SOC = Sense Of Coherence; QoL = Quality of Life.

#### Anxiety

There was no statistical evidence for a difference in evolution of the sSTAI-6 between intervention and control arm, nor between the different pre-pregnancy BMI categories. The sSTAI-6 increases in the first year after childbirth, with a steeper increase between baseline and end of intervention (+1.97 (95% CI, 1.14 to 2.79)) as compared to the 6 months follow-up (+0.75 (95% CI, -0.17 to 1.67)). Higher values of sSTAI-6 were associated to lower QoL at baseline (+3.6 per 10 pts decrease (95% CI, 3.1 to 4.0)) and more excessive weight (+0.16 (95% CI, 0.03 to 0.30) per 1 kg increase in excessive weight).

**Fig 2:**
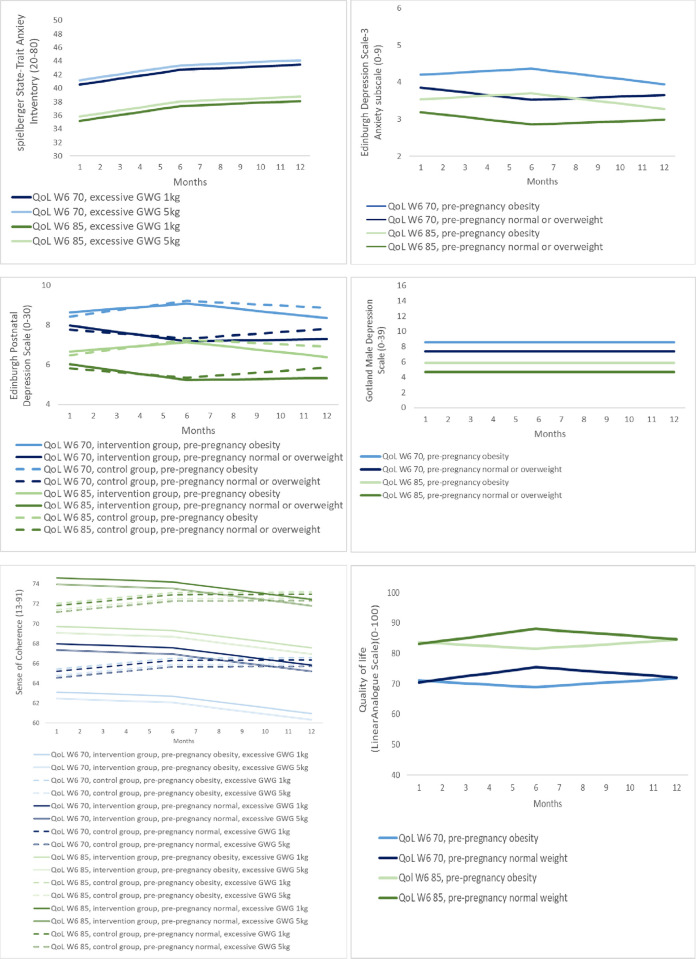
Effect of the INTER-ACT lifestyle intervention on the evolution of mental health (mixed models). QoL = Quality of Life; W6 = week 6; GWG = gestational weight gain.

The evolution in EDS3-A did not seem to differ between intervention and control arm, but differed by pre-pregnancy BMI. Women with pre-pregnancy obesity had an increase between baseline and end of intervention (+0.15 (95%CI, -0.25 to 0.54)), followed by a decrease till 6 months follow-up (-0.42 (95%CI, -0.83 to -0.004)). While women with a normal/overweight pre-pregnancy BMI showed a decrease in EDS-3A between baseline and end of intervention (-0.30 (95% CI, -0.46 to -0.14)) and increase afterwards (+0.13 (95% CI, -0.03 to 0.29) between end of intervention and 6 months follow-up). Higher values of EDS3-A were seen in women with lower QoL at baseline (+0.4 (95% CI, 0.36 to 0.53) per 10 pts decrease) (Table 4, S1 File and Fig 2).

#### Depression

The evolution in EPDS differed between the intervention and control arm, depending on whether the woman had pre-pregnancy obesity or not. Women with pre-pregnancy obesity showed an increase in EPDS between baseline and end of intervention, but decrease afterwards: in the control arm+ 0.71 (95% CI, -0.12 to 1.54) between baseline and end of intervention, and -0.35 (95% CI, -1.25 to 0.56) till 6 months follow-up; in the intervention arm + 0.42 (95% CI -0.42 to 1.25) between baseline and end of intervention, and– 0.74 (95% CI, -1.64 to 0.15) till 6 months follow-up. Women with normal/overweight pre-pregnancy BMI, reported a decrease between baseline and end of intervention, followed by an increase till 6 months follow-up: in the control arm -0.42 (95% CI, -0.76 to -0.08) from baseline till end of intervention and +0.50 (95% CI, 0.10 to 0.90) till 6 months follow-up; in the intervention arm -0.71 (95% CI, -1.07 to -0.35) from baseline till end of intervention and +0.10 (-0.26 to 0.47) till 6 months follow-up.

Higher values of EPDS were seen in women with lower QoL at baseline (+1.3 (95% CI 1.12 to 1.48) per 10 pts decrease).

No statistical evidence was found for an evolution in GMDS during the first year (Table 4, S1 File and Fig 2).

#### Sense of coherence

The evolution of SOC during the first year after childbirth depended on whether or not women received the INTER-ACT intervention, her pre-pregnancy BMI, her QoL at baseline, and the amount of excessive GWG.

Women in the intervention arm showed a decrease in SOC over time, but only in women with a normal or obese pre-pregnancy BMI: -0.36 (95% CI, -1.60 to 0.88) between baseline and end of intervention, -0.20 (95% CI, -1.43 to 1.04) between end of intervention and 6 months follow-up. In women with overweight pre-pregnancy (in the intervention arm): +1.53 (95% CI, -0.08 to 3.15) between baseline and end of intervention; stable afterwards (+0.06 (95% CI, -1.59 to 1.70) between end of intervention and 6 months follow-up).

In the control arm, the values of SOC seemed to be increasing during the first year, again only in women with a normal/obese pre-pregnancy BMI: increase of +0.98 (95% CI, -0.13 to 2.09) between baseline and end of intervention, stable between end of intervention and 6 months follow-up (+0.05 (95% CI, -1.22 to 1.32)). In women with overweight pre-pregnancy in the control arm, there seemed to be a decrease of -1.77 (95% CI, -3.32 to -0.22) between baseline and end of intervention, followed by an increase of +0.56 (95% CI, -1.29 to 2.41) between end of intervention and 6 months follow-up.

Higher values of SOC were associated to higher QoL at baseline (+4.4 (95% CI, 3.93 to 4.89) per 10 pts increase) and less excessive weight (+0.16 (95% CI, 0.008 to 0.31) per 1 kg decrease in excessive weight) (Table 4, S1 File and Fig 2).

#### Quality of life

There was no statistical evidence for a difference in evolution of QoL between the intervention arm and the control arm. But the evolution in QoL differed by pre-pregnancy BMI: Women with obese pre-pregnancy BMI started with the highest reported QoL (+2.04 (95% CI, 0.75 to 3.33)) (as compared no normal weight), but had a decrease in QOL (-2.01 (95% CI, -4.25 to 0.23)) between baseline and end of intervention, and increase of +3.06 (95% CI, 0.55 to 5.57) till 6 months follow-up. Women with overweight pre-pregnancy start also with a higher reported QoL (+1.48 (95% CI, 0.53 to 2.42)), but their QoL almost does not change: +0.43 (95% CI, -0.98 to 1.85) between baseline and end of intervention, and -0.33 (95% CI, -1.87 to 1.22) between end of intervention and 6 months follow-up. Women with normal pre-pregnancy BMI had an increase +4.46 (95% CI, 3.32 to 5.59) from baseline till end of intervention, followed by a decrease of -3.35 (95% CI, -4.59 to -2.11) till 6 months follow-up (Table 4, S1 File and Fig 2).

## Discussion

To our knowledge, this is the first RCT evaluating the effect of a postpartum lifestyle intervention, including a mental health component on levels of anxiety, depression, SOC and QoL during the first year after childbirth in women with pre-existing excessive GWG. We found that the INTER-ACT lifestyle intervention was associated with an improvement in the number of women with depressive symptoms, or low SOC at 6 months after intervention, but only in normal weight women. Mixed models revealed that both our intervention as well as the pre-pregnancy BMI category and QoL at baseline were associated with levels of depressive symptoms throughout the first year after childbirth. Women with pre-pregnancy obesity showed an increase in EPDS between baseline and end of intervention, but an decrease afterwards. Women with normal/overweight pre-pregnancy BMI showed a significant decrease in depressive symptoms between baseline and end of intervention, followed by an increase till 6 months follow-up. A higher QoL at baseline had a significant positive effect on levels of depression, mainly in women with pre-pregnancy normal weight and overweight. The evolution of SOC depended on pre-pregnancy BMI, the amount of excessive GWG and the QoL at 6 weeks after childbirth. Women with a normal pre-pregnancy BMI reported better levels of SOC, compared to normal weight women of the control arm. Women with pre-pregnancy obesity reported worser levels of SOC if they received the INTER-ACT intervention compared to the control arm. Lower levels of excessive GWG (1kg vs 5kg) and a higher QoL at baseline had a significant positive effect on SOC.

Previous studies focusing on lifestyle interventions in the general population showed that women with obesity experience a lack of motivation, and therefore less adherence to treatments [35, 36]. Our results are in line with this. Our population of women with a normal pre-pregnancy BMI were more adherent to complete the intervention than women with overweight or obesity (27% vs 33 and 42% respectively). However, our results revealed new findings in the difference between women with normal/ overweight and women with obesity after the end of intervention, (6 months follow-up). Women with pre-pregnancy obesity reported a decrease in depressive symptoms in the 6 months after intervention, compared to women with pre-pregnancy normal/overweight who reported an increase in depressive symptoms during the 6 months after intervention. Although obese women who received the INTER-ACT intervention showed higher levels of depressive symptoms during the intervention, their long-term outcomes improved. A possible reason for the deterioration of depressive symptoms in women with obesity during the intervention period can be found in the fact that the focus on weight and weight loss has an initial adverse effect on mood and subsequently motivation in women with existing obesity. Further RCT’s are needed to investigate which interventions are effective to support women with obesity in their empowerment and self-efficacy. Moreover, our results showed that pre-pregnancy BMI still has an important role in the evolution of anxiety, depression, SOC and QoL, with differences in evolution between women with normal/overweight pre-pregnancy and women with pre-pregnancy obesity. This suggests that postpartum follow-up in the context of mental health requires an individual approach for each BMI group. And most important, lifestyle interventions that target weight should be integrated into preconception care to prevent long-term mental health problems.

Anxiety (sSTAI-6) increased over time. These findings are in line with our previous research [2]. However, current results reveal that the increase in anxiety during the first year after childbirth depends on QoL at 6 weeks postpartum and women’s weight (sSTAI-6: excessive GWG; EDS-3A: pre-pregnancy BMI). Moreover, QoL at 6 weeks after childbirth appears to be a contributing factor within the evolution of anxiety, depression, SOC and QoL first year after childbirth. The QoL was scored using one comprehensive score that summarized physical, psychological and social aspects. QoL screening at 6 weeks after childbirth, should be integrated into standard care pathways, using 1 comprehensive question, to contribute to early detection of mental health problems in the first year postpartum and targeted follow-up.

The mental health component of the INTER-ACT intervention focused on stressors and social support. Lack of social support is often identified as a major cause of adverse mental health [37]. Studies focusing on social support showed mixed results in terms of effectiveness on mental health outcomes [38]. In addition, there is only evidence for a short-term effectiveness on social support [39, 40]. This may indicate that in the future we will have to adjust the focus of the mental health component and add other evidence-based interventions known for their effect on resilience/overall mental well-being (e.g. interpersonal therapy [41], mindfulness-based interventions [42–44]). Systematic reviews and meta-analyses of mindfulness-based interventions in the perinatal period found evidence of reductions in stress, anxiety and depression from pre-post studies [42–44] with larger effect-sizes for studies on participants with a history of depression, anxiety and stress [45, 46]. A more recent review and meta-analysis, based on 12 controlled trials on women without pre-existing disorders, showed small but clear reductions in symptoms of depression [47]. The use of a present-focused short-term therapy with emphasis on the interpersonal context (interpersonal therapy) has been adapted in previous research for the treatment of women during the perinatal period and has proven to be an effective strategy for the prevention of PPD [39].

The fact that the INTER-ACT intervention is limited to social support and stressors, and we did not select women based on the presence of mental health problems, may be a reason for the absence of large effects of mental health improvement after receiving the INTER-ACT intervention. Indeed, as shown in Table 2, the women in our sample proved a median score of 6 for EPDS, 5 for GMDS, 37 for s STAI-6, 4 for EDS-3A, 80 for QoL and 70 for SOC-13, that shows that our population is provided with a number of women with a relative good mental health at baseline.

Stress and depression appear to be predictors of poor adherence to lifestyle interventions [48]. Regardless of the presence of a number of women with relative good mental health scores at baseline, our previous research showed that nearly half of the studied population of women struggle with symptoms of depression, anxiety, low SOC and low QoL at 6 weeks after childbirth [2]. Because of this, they may have less interest or motivation to adhere to the INTER-ACT lifestyle intervention. During the postpartum period, evidence-based psychological interventions are therefore recommended in combination with diet-and exercise/physical-based interventions in the treatment of poor maternal mental health. For example: cognitive behaviour therapy, mindfulness-based interventions, interpersonal therapy and professionally home-based visits.

The strengths of this study were the study design (a multicentre RCT with long-term follow-up), the large study group and the analysis of four different mental health outcomes. Nevertheless, there were also some limitations in this study. The mental health questionnaires were completed prior to the coaching session, so the effect of the coaching session at that time point was not yet apparent in the mental health outcomes. A second limitation is that we presume that our control arm may have been triggered by the regular home visits they received as part of the data-collection (anthropometrics). During the measurements conversations on diet, physical activity and mental well-being were not initiated, however, we believe that by home visits and interaction with mothers, a trigger may have occurred that can have an effect on the mental well-being. A third limitation is that our results cannot be fully generalized to the entire population of postpartum women as our sample mainly consisted of working women (92%) who held at least a bachelor’s degree (71%). A fourth limitation is that within the INTER-ACT trial, power analysis was driven solely by the primary outcome: a composite outcome of occurrence of pregnancy- and birth related problems (pregnancy induced hypertension, gestational diabetes, caesarean section and large for gestational age babies). As a result, there may be a lack of statistical power in current analyses that focus on mental health outcomes, especially in the pre-pregnant obese women. This might limit the statistical evidence of the inter-act intervention on mental health, but can induce as well rather chance findings such as e.g. the significant difference in GMDS between the intervention and control arm at 6 months follow-up in the normal-weight women.

## Conclusion

The INTER-ACT postpartum lifestyle intervention shows improvement in depressive symptoms and levels of SOC, with differences within pre-pregnancy BMI. Attention to women’s QoL at baseline and pre-pregnancy weight are important key factors for maternal mental health the first year after childbirth. In addition, women in the postpartum period may benefit more from lifestyle interventions if they also include evidence-based psychological approaches, both in terms of addressing mental health as well as addressing the adherence needed to create a long-term effect.

## Supporting information

S1 Checklist CONSORT 2010 checklist of information to include when reporting a randomised trial*. (PDF)

S1 File Explanation of variables (mixed models SAS output). (PDF)

S1 Protocol (PDF)
