# Supplementary material for: Effect of the INTER-ACT lifestyle intervention on maternal mental health during the first year after childbirth: A randomized controlled trial
Source: PLoS One. 2023 Jul 28;18(7):e0284770. doi: 10.1371/journal.pone.0284770 (PMC10381046; doi:10.1371/journal.pone.0284770)
Supplement: S1 File — (PDF) [file pone.0284770.s002.pdf]

Explanation of variables (Mixed Models SAS output)

**Outcome variables**	**Explanation**
sSTAI	Spielberger State-Trait Anxiety Inventory<br>Assessed anxiety<br>Continuous variable
EDS-3A	Edinburgh Depression Scale – 3 Anxiety subscale<br>Assessed anxiety<br>Continuous variable
EPDS	Edinburgh Postnatal Depression Scale<br>Assessed depression<br>Continuous variable
GMDS	Gotland Male Depression Scale<br>Assessed depression<br>Continuous variable
SOC	Sense Of Coherence<br>Assessed sense of coherence<br>Continuous variable
QoL	Quality of Life<br>Assessed quality of life<br>Continuous variable

**Explanatory variables**	**Explanation**
Months	Months since childbirth
M6	1 if month >6, 0 if months <6), in order to model a breakpoint at month 6.
MonthsMinus6	months - 6
Overweight	1 if pre-pregnancy BMI between 25 and 29.9, 0 otherwise
Obese	1 if pre-pregnancy BMI>=30, 0 otherwise
Intervention	1=intervention arm, 0=control arm
W6QoL	Quality of Life at week 6
Exc	Amount of excessive gestational weight gain above the IOM cutoff, depending on the pre-pregnancy BMI (above 16kg for normal pre-pregnancy BMI, above 11.5 kg for overweight, and above 9kg for obese pre-pregnancy BMI)

Spielberger State-Trait Anxiety Inventory (sSTAI)

The SAS System

The Mixed Procedure

Model Information	
**Data Set**	WORK.B
**Dependent Variable**	sSTAI
**Covariance Structure**	Unstructured
**Subject Effect**	Record_Id
**Estimation Method**	REML
**Residual Variance Method**	None
**Fixed Effects SE Method**	Model-Based
**Degrees of Freedom Method**	Between-Within

Spielberger State-Trait Anxiety Inventory (sSTAI)

The SAS System

The Mixed Procedure

Class Level Information		
**Class**	**Levels**	**Values**

Spielberger State-Trait Anxiety Inventory (sSTAI)

The SAS System

The Mixed Procedure

Class Level Information		
**Class**	**Levels**	**Values**
**Record_Id**	1044	AUG001 AUG002 AUG003 AUG004 AUG006 AUG007 AUG008 AUG009 AUG011 AUG012 AUG013 AUG014 AUG015 AUG016 AUG017 AUG019 AUG020 AUG021 AUG022 AUG024 AUG025 AUG026 AUG027 AUG028 AUG029 AUG030 AUG031 AUG033 AUG034 AUG035 AUG036 AUG037 AUG038 AUG039 AUG040 AUG041 AUG042 AUG043 AUG044 AUG045 AUG046 AUG047 AUG048 AUG049 AUG050 AUG051 AUG052 AUG053 AUG057 AUG058 AUG059 AUG061 AUG063 AUG064 AUG065 AUG066 AUG067 AUG068 AUG069 AUG070 AUG071 AUG080 AUG081 AUG083 AUG087 AUG089 AUG090 AUG095 AUG096 AUG097 AUG100 AUG101 AUG103 AUG104 AUG105 AUG107 AUG109 AUG110 AUG111 AUG113 AUG115 AUG116 AUG117 AUG127 AUG129 AUG130 AUG132 AUG135 AUG137 AUG138 AUG141 AUG143 AUG145 AUG147 AUG148 AUG150 AUG151 AUG152 AUG153 AUG155 AUG157 AUG158 AUG160 AUG161 AUG162 AUG164 AUG166 AUG169 AUG171 AUG172 AUG174 AUG176 AUG178 AUG179 AUG180 AUG181 AUG182 AUG184 AUG185 AUG186 AUG187 AUG188 AUG191 AUG193 AUG198 AUG201 AUG202 AUG203 AUG204 HEU001 HEU003 HEU004 HEU006 HEU007 HEU009 HEU010 HEU012 HEU013 HEU014 HEU015 HEU016 HEU017 HEU018 HEU019 HEU020 HEU021 HEU022 HEU023 HEU024 HEU025 HEU026 HEU027 HEU028 HEU029 HEU030 HEU031 HEU034 HEU035 HEU038 HEU040 HEU041 HEU042 HEU044 HEU045 HEU046 HEU047 HEU048 HEU049 HEU050 HEU051 HEU052 HEU054 HEU055 HEU056 HEU057 HEU058 HEU059 HEU060 HEU062 HEU063 HEU064 HEU065 HEU066 HEU067 HEU068 HEU069 HEU071 HEU072 HEU073 HEU074 HEU075 HEU076 HEU077 HEU078 HEU079 HEU081 HEU082 HEU083 HEU084 HEU085 HEU086 HEU087 HEU088 HEU089 HEU090 HEU091 HEU093 HEU095 HEU097 HEU098 HEU100 HEU102 HEU103 HEU104 HEU105 HEU106 HEU107 HEU108 HEU109 HEU110 HEU112 HEU113 HEU114 HEU115 HEU116 HEU117 HEU118 HEU119 HEU120 HEU121 HEU122 HEU123 HEU125 HEU126 HEU127 HEU128 HEU129 HEU130 HEU131 HEU133 HEU135 HEU136 HEU137 JES001 JES003 JES004 JES006 JES008 JES009 JES010 JES011 JES012 JES013 JES014 JES017 JES018 JES020 JES022 JES024 JES025 JES027 JES028 JES029 JES030 JES032 JES033 JES035 JES036 JES037 JES038 JES040 JES041 JES042 JES043 JES045 JES047 JES048 JES049 JES050 JES051 JES053 JES054 JES055 JES056 JES058 JES060 JES063 JES066 JES067 JES069 JES070 JES071 JES073 JES074 JES076 JES077 JES078 JES079 JES080 JES083 JES085 JES086 JES088

Spielberger State-Trait Anxiety Inventory (sSTAI)

The SAS System

The Mixed Procedure

Dimensions	
**Covariance Parameters**	10
**Columns in X**	5
**Columns in Z**	0
**Subjects**	1044
**Max Obs per Subject**	4

Number of Observations	
**Number of Observations Read**	2716
**Number of Observations Used**	2673
**Number of Observations Not Used**	43

Iteration History			
**Iteration**	**Evaluations**	**-2 Res Log Like**	**Criterion**
**0**	1	20153.94146363	
**1**	2	19651.35277668	0.00001914
**2**	1	19651.20697261	0.00000006
**3**	1	19651.20654589	0.00000000

Convergence criteria met.

Spielberger State-Trait Anxiety Inventory (sSTAI)

The SAS System

The Mixed Procedure

Estimated R Matrix for Record_Id AUG001	
**Row**	**Col1**
**1**	97.3996

Covariance Parameter Estimates					
**Cov Parm**	**Subject**	**Estimate**	**Standard Error**	**Z Value**	**Pr Z**
**UN(1,1)**	Record_Id	97.3996	4.2747	22.78	<.0001
**UN(2,1)**	Record_Id	48.5672	3.9444	12.31	<.0001
**UN(2,2)**	Record_Id	105.90	5.4252	19.52	<.0001
**UN(3,1)**	Record_Id	47.8144	4.7100	10.15	<.0001
**UN(3,2)**	Record_Id	57.0434	5.1043	11.18	<.0001
**UN(3,3)**	Record_Id	132.05	7.5218	17.56	<.0001
**UN(4,1)**	Record_Id	44.1321	6.6472	6.64	<.0001
**UN(4,2)**	Record_Id	59.2452	7.6387	7.76	<.0001
**UN(4,3)**	Record_Id	72.5440	8.1672	8.88	<.0001
**UN(4,4)**	Record_Id	133.79	12.1139	11.04	<.0001

Spielberger State-Trait Anxiety Inventory (sSTAI)

The SAS System

The Mixed Procedure

Asymptotic Covariance Matrix of Estimates											
**Row**	**Cov Parm**	**CovP1**	**CovP2**	**CovP3**	**CovP4**	**CovP5**	**CovP6**	**CovP7**	**CovP8**	**CovP9**	**CovP10**
**1**	UN(1,1)	18.2733	9.0866	4.4758	8.9435	4.4116	4.3314	8.2481	4.0628	3.9843	3.6667
**2**	UN(2,1)	9.0866	15.5581	13.3032	8.7984	9.8534	6.4425	8.9835	9.5565	6.4215	6.3315
**3**	UN(2,2)	4.4758	13.3032	29.4332	6.5539	15.7282	8.1636	6.9367	16.3546	8.5878	8.9344
**4**	UN(3,1)	8.9435	8.7984	6.5539	22.1842	13.2946	19.7019	12.0386	8.1091	14.0094	9.2011
**5**	UN(3,2)	4.4116	9.8534	15.7282	13.2946	26.0538	23.3500	8.4420	15.6881	18.1233	12.5979
**6**	UN(3,3)	4.3314	6.4425	8.1636	19.7019	23.3500	56.5781	9.8618	12.1361	30.6613	16.4979
**7**	UN(4,1)	8.2481	8.9835	6.9367	12.0386	8.4420	9.8618	44.1851	25.1754	27.4281	40.4784
**8**	UN(4,2)	4.0628	9.5565	16.3546	8.1091	15.6881	12.1361	25.1754	58.3499	35.5269	59.0365
**9**	UN(4,3)	3.9843	6.4215	8.5878	14.0094	18.1233	30.6613	27.4281	35.5269	66.7030	67.0398
**10**	UN(4,4)	3.6667	6.3315	8.9344	9.2011	12.5979	16.4979	40.4784	59.0365	67.0398	146.75

Fit Statistics	
**-2 Res Log Likelihood**	19651.2
**AIC (Smaller is Better)**	19671.2
**AICC (Smaller is Better)**	19671.3
**BIC (Smaller is Better)**	19720.7

Spielberger State-Trait Anxiety Inventory (sSTAI)

The SAS System

The Mixed Procedure

Null Model Likelihood Ratio Test		
DF	Chi-Square	Pr > ChiSq
9	502.73	<.0001

Solution for Fixed Effects					
Effect	Estimate	Standard Error	DF	t Value	Pr > |t|
Intercept	64.8726	1.7622	1041	36.81	<.0001
months	0.4370	0.09363	1041	4.67	<.0001
W6LAS	-0.3563	0.02170	1041	-16.42	<.0001
exc	0.1644	0.06778	1041	2.43	0.0155
M6*monthsMinus6	-0.3121	0.1470	1041	-2.12	0.0340

Type 3 Tests of Fixed Effects				
Effect	Num DF	Den DF	F Value	Pr > F
months	1	1041	21.79	<.0001
W6LAS	1	1041	269.69	<.0001
exc	1	1041	5.88	0.0155
M6*monthsMinus6	1	1041	4.51	0.0340

Edinburgh Depression Scale- 3 Anxiety subscale (EDS-3A)

The SAS System

The Mixed Procedure

Model Information	
**Data Set**	WORK.B
**Dependent Variable**	EDS3A
**Covariance Structure**	Unstructured
**Subject Effect**	Record_Id
**Estimation Method**	REML
**Residual Variance Method**	None
**Fixed Effects SE Method**	Model-Based
**Degrees of Freedom Method**	Between-Within

Edinburgh Depression Scale- 3 Anxiety subscale (EDS-3A)

The SAS System

The Mixed Procedure

Class Level Information		
**Class**	**Levels**	**Values**

Edinburgh Depression Scale- 3 Anxiety subscale (EDS-3A)

The SAS System

The Mixed Procedure

Class Level Information		
**Class**	**Levels**	**Values**
**Record_Id**	1044	AUG001 AUG002 AUG003 AUG004 AUG006 AUG007 AUG008 AUG009 AUG011 AUG012 AUG013 AUG014 AUG015 AUG016 AUG017 AUG019 AUG020 AUG021 AUG022 AUG024 AUG025 AUG026 AUG027 AUG028 AUG029 AUG030 AUG031 AUG033 AUG034 AUG035 AUG036 AUG037 AUG038 AUG039 AUG040 AUG041 AUG042 AUG043 AUG044 AUG045 AUG046 AUG047 AUG048 AUG049 AUG050 AUG051 AUG052 AUG053 AUG057 AUG058 AUG059 AUG061 AUG063 AUG064 AUG065 AUG066 AUG067 AUG068 AUG069 AUG070 AUG071 AUG080 AUG081 AUG083 AUG087 AUG089 AUG090 AUG095 AUG096 AUG097 AUG100 AUG101 AUG103 AUG104 AUG105 AUG107 AUG109 AUG110 AUG111 AUG113 AUG115 AUG116 AUG117 AUG127 AUG129 AUG130 AUG132 AUG135 AUG137 AUG138 AUG141 AUG143 AUG145 AUG147 AUG148 AUG150 AUG151 AUG152 AUG153 AUG155 AUG157 AUG158 AUG160 AUG161 AUG162 AUG164 AUG166 AUG169 AUG171 AUG172 AUG174 AUG176 AUG178 AUG179 AUG180 AUG181 AUG182 AUG184 AUG185 AUG186 AUG187 AUG188 AUG191 AUG193 AUG198 AUG201 AUG202 AUG203 AUG204 HEU001 HEU003 HEU004 HEU006 HEU007 HEU009 HEU010 HEU012 HEU013 HEU014 HEU015 HEU016 HEU017 HEU018 HEU019 HEU020 HEU021 HEU022 HEU023 HEU024 HEU025 HEU026 HEU027 HEU028 HEU029 HEU030 HEU031 HEU034 HEU035 HEU038 HEU040 HEU041 HEU042 HEU044 HEU045 HEU046 HEU047 HEU048 HEU049 HEU050 HEU051 HEU052 HEU054 HEU055 HEU056 HEU057 HEU058 HEU059 HEU060 HEU062 HEU063 HEU064 HEU065 HEU066 HEU067 HEU068 HEU069 HEU071 HEU072 HEU073 HEU074 HEU075 HEU076 HEU077 HEU078 HEU079 HEU081 HEU082 HEU083 HEU084 HEU085 HEU086 HEU087 HEU088 HEU089 HEU090 HEU091 HEU093 HEU095 HEU097 HEU098 HEU100 HEU102 HEU103 HEU104 HEU105 HEU106 HEU107 HEU108 HEU109 HEU110 HEU112 HEU113 HEU114 HEU115 HEU116 HEU117 HEU118 HEU119 HEU120 HEU121 HEU122 HEU123 HEU125 HEU126 HEU127 HEU128 HEU129 HEU130 HEU131 HEU133 HEU135 HEU136 HEU137 JES001 JES003 JES004 JES006 JES008 JES009 JES010 JES011 JES012 JES013 JES014 JES017 JES018 JES020 JES022 JES024 JES025 JES027 JES028 JES029 JES030 JES032 JES033 JES035 JES036 JES037 JES038 JES040 JES041 JES042 JES043 JES045 JES047 JES048 JES049 JES050 JES051 JES053 JES054 JES055 JES056 JES058 JES060 JES063 JES066 JES067 JES069 JES070 JES071 JES073 JES074 JES076 JES077 JES078 JES079 JES080 JES083 JES085 JES086 JES088

Edinburgh Depression Scale- 3 Anxiety subscale (EDS-3A)

The SAS System

The Mixed Procedure

Dimensions	
**Covariance Parameters**	10
**Columns in X**	7
**Columns in Z**	0
**Subjects**	1044
**Max Obs per Subject**	4

Number of Observations	
**Number of Observations Read**	2716
**Number of Observations Used**	2673
**Number of Observations Not Used**	43

Iteration History

Iteration	Evaluations	-2 Res Log Like	Criterion
**0**	1	11446.80913470	
**1**	2	10670.63190547	0.00002461
**2**	1	10670.55925020	0.00000003
**3**	1	10670.55915036	0.00000000

Convergence criteria met.

Edinburgh Depression Scale- 3 Anxiety subscale (EDS-3A)

The SAS System

The Mixed Procedure

Estimated R Matrix for Record_Id AUG001	
**Row**	**Col1**
**1**	4.3063

Covariance Parameter Estimates					
**Cov Parm**	**Subject**	**Estimate**	**Standard Error**	**Z Value**	**Pr Z**
**UN(1,1)**	Record_Id	4.3063	0.1888	22.81	<.0001
**UN(2,1)**	Record_Id	2.3738	0.1595	14.88	<.0001
**UN(2,2)**	Record_Id	3.9136	0.1968	19.89	<.0001
**UN(3,1)**	Record_Id	2.3561	0.1803	13.07	<.0001
**UN(3,2)**	Record_Id	2.3403	0.1802	12.99	<.0001
**UN(3,3)**	Record_Id	4.4140	0.2475	17.84	<.0001
**UN(4,1)**	Record_Id	2.2403	0.2302	9.73	<.0001
**UN(4,2)**	Record_Id	2.3348	0.2323	10.05	<.0001
**UN(4,3)**	Record_Id	2.8611	0.2494	11.47	<.0001
**UN(4,4)**	Record_Id	4.2098	0.3487	12.07	<.0001

Edinburgh Depression Scale- 3 Anxiety subscale (EDS-3A)

The SAS System

The Mixed Procedure

Asymptotic Covariance Matrix of Estimates											
**Row**	**Cov Parm**	**CovP1**	**CovP2**	**CovP3**	**CovP4**	**CovP5**	**CovP6**	**CovP7**	**CovP8**	**CovP9**	**CovP10**
**1**	UN(1,1)	0.03565	0.01965	0.01081	0.01951	0.01073	0.01065	0.01852	0.01018	0.01010	0.009576
**2**	UN(2,1)	0.01965	0.02544	0.02215	0.01647	0.01716	0.01212	0.01626	0.01668	0.01189	0.01161
**3**	UN(2,2)	0.01081	0.02215	0.03872	0.01222	0.02266	0.01287	0.01230	0.02268	0.01294	0.01293
**4**	UN(3,1)	0.01951	0.01647	0.01222	0.03251	0.02123	0.02980	0.02110	0.01478	0.02303	0.01696
**5**	UN(3,2)	0.01073	0.01716	0.02266	0.02123	0.03248	0.03033	0.01501	0.02235	0.02431	0.01840
**6**	UN(3,3)	0.01065	0.01212	0.01287	0.02980	0.03033	0.06125	0.01764	0.01831	0.03881	0.02426
**7**	UN(4,1)	0.01852	0.01626	0.01230	0.02110	0.01501	0.01764	0.05301	0.03290	0.03561	0.05114
**8**	UN(4,2)	0.01018	0.01668	0.02268	0.01478	0.02235	0.01831	0.03290	0.05396	0.03534	0.05387
**9**	UN(4,3)	0.01010	0.01189	0.01294	0.02303	0.02431	0.03881	0.03561	0.03534	0.06220	0.06493
**10**	UN(4,4)	0.009576	0.01161	0.01293	0.01696	0.01840	0.02426	0.05114	0.05387	0.06493	0.1216

Fit Statistics	
**-2 Res Log Likelihood**	10670.6
**AIC (Smaller is Better)**	10690.6
**AICC (Smaller is Better)**	10690.6
**BIC (Smaller is Better)**	10740.1

Edinburgh Depression Scale- 3 Anxiety subscale (EDS-3A)

The SAS System

The Mixed Procedure

Null Model Likelihood Ratio Test		
DF	Chi-Square	Pr > ChiSq
9	776.25	<.0001

Solution for Fixed Effects					
Effect	Estimate	Standard Error	DF	t Value	Pr > |t|
Intercept	7.0263	0.3537	1041	19.87	<.0001
months	-0.06670	0.01779	1041	-3.75	0.0002
obese	0.2467	0.2179	1041	1.13	0.2579
W6LAS	-0.04435	0.004474	1041	-9.91	<.0001
months*obese	0.09925	0.04793	1041	2.07	0.0386
M6*monthsMinus6	0.08819	0.02707	1041	3.26	0.0012
obese*M6*monthsMinus	-0.1904	0.07327	1041	-2.60	0.0095

Type 3 Tests of Fixed Effects				
Effect	Num DF	Den DF	F Value	Pr > F
months	1	1041	14.06	0.0002
obese	1	1041	1.28	0.2579
W6LAS	1	1041	98.26	<.0001
months*obese	1	1041	4.29	0.0386

Edinburgh Depression Scale- 3 Anxiety subscale (EDS-3A)

The SAS System

The Mixed Procedure

Type 3 Tests of Fixed Effects				
**Effect**	**Num DF**	**Den DF**	**F Value**	**Pr > F**
**M6*monthsMinus6**	1	1041	10.61	0.0012
**obese*M6*monthsMinus**	1	1041	6.75	0.0095

Edinburgh Postnatal Depression Scale (EPDS)

The SAS System

The Mixed Procedure

Model Information	
**Data Set**	WORK.B
**Dependent Variable**	EPDS
**Covariance Structure**	Unstructured
**Subject Effect**	Record_Id
**Estimation Method**	REML
**Residual Variance Method**	None
**Fixed Effects SE Method**	Model-Based
**Degrees of Freedom Method**	Between-Within

Edinburgh Postnatal Depression Scale (EPDS)

The SAS System

The Mixed Procedure

Class Level Information		
**Class**	**Levels**	**Values**

Edinburgh Postnatal Depression Scale (EPDS)

The SAS System

The Mixed Procedure

Class Level Information		
**Class**	**Levels**	**Values**
**Record_Id**	1037	AUG001 AUG002 AUG003 AUG004 AUG006 AUG007 AUG008 AUG009 AUG011 AUG012 AUG013 AUG014 AUG015 AUG016 AUG017 AUG019 AUG020 AUG021 AUG022 AUG024 AUG025 AUG026 AUG027 AUG028 AUG029 AUG030 AUG031 AUG033 AUG034 AUG035 AUG036 AUG037 AUG038 AUG039 AUG040 AUG041 AUG042 AUG043 AUG044 AUG045 AUG046 AUG047 AUG048 AUG049 AUG050 AUG051 AUG052 AUG053 AUG057 AUG058 AUG059 AUG061 AUG063 AUG064 AUG065 AUG066 AUG067 AUG068 AUG069 AUG070 AUG071 AUG080 AUG081 AUG083 AUG087 AUG089 AUG090 AUG095 AUG096 AUG097 AUG100 AUG101 AUG103 AUG104 AUG105 AUG107 AUG109 AUG110 AUG111 AUG113 AUG115 AUG116 AUG117 AUG127 AUG129 AUG130 AUG132 AUG135 AUG137 AUG138 AUG141 AUG143 AUG145 AUG147 AUG148 AUG150 AUG151 AUG152 AUG153 AUG155 AUG157 AUG158 AUG160 AUG161 AUG162 AUG164 AUG166 AUG169 AUG171 AUG172 AUG174 AUG176 AUG178 AUG179 AUG180 AUG181 AUG182 AUG184 AUG185 AUG186 AUG187 AUG188 AUG191 AUG193 AUG198 AUG201 AUG202 AUG203 AUG204 HEU001 HEU003 HEU004 HEU006 HEU007 HEU009 HEU010 HEU012 HEU013 HEU014 HEU015 HEU016 HEU017 HEU018 HEU019 HEU020 HEU021 HEU022 HEU023 HEU024 HEU025 HEU026 HEU027 HEU028 HEU029 HEU030 HEU031 HEU034 HEU035 HEU038 HEU040 HEU041 HEU042 HEU044 HEU045 HEU046 HEU047 HEU048 HEU049 HEU050 HEU051 HEU052 HEU054 HEU055 HEU056 HEU057 HEU058 HEU059 HEU060 HEU062 HEU063 HEU064 HEU065 HEU066 HEU067 HEU068 HEU069 HEU071 HEU072 HEU073 HEU074 HEU075 HEU076 HEU077 HEU078 HEU079 HEU081 HEU082 HEU083 HEU084 HEU085 HEU086 HEU087 HEU088 HEU089 HEU090 HEU091 HEU093 HEU095 HEU097 HEU098 HEU100 HEU102 HEU103 HEU104 HEU105 HEU106 HEU107 HEU108 HEU109 HEU110 HEU112 HEU113 HEU114 HEU115 HEU116 HEU117 HEU118 HEU119 HEU120 HEU121 HEU122 HEU123 HEU125 HEU126 HEU127 HEU128 HEU129 HEU130 HEU131 HEU133 HEU135 HEU136 HEU137 JES001 JES003 JES004 JES006 JES008 JES009 JES010 JES011 JES012 JES013 JES014 JES017 JES018 JES020 JES022 JES024 JES025 JES027 JES028 JES029 JES030 JES032 JES033 JES035 JES036 JES037 JES038 JES040 JES041 JES042 JES043 JES045 JES047 JES048 JES049 JES050 JES051 JES053 JES054 JES055 JES056 JES060 JES063 JES066 JES067 JES069 JES070 JES071 JES073 JES074 JES076 JES077 JES078 JES079 JES080 JES083 JES085 JES086 JES088 JES091

Edinburgh Postnatal Depression Scale (EPDS)

The SAS System

The Mixed Procedure

Dimensions	
**Covariance Parameters**	10
**Columns in X**	9
**Columns in Z**	0
**Subjects**	1037
**Max Obs per Subject**	4

Number of Observations	
**Number of Observations Read**	2671
**Number of Observations Used**	2629
**Number of Observations Not Used**	42

Iteration History			
**Iteration**	**Evaluations**	**-2 Res Log Like**	**Criterion**
**0**	1	14929.01643934	
**1**	2	14216.36536800	0.00004133
**2**	1	14216.16337375	0.00000018
**3**	1	14216.16251258	0.00000000

Convergence criteria met.

Edinburgh Postnatal Depression Scale (EPDS)

The SAS System

The Mixed Procedure

Estimated R Matrix for Record_Id AUG001	
**Row**	**Col1**
**1**	16.6788

Covariance Parameter Estimates					
**Cov Parm**	**Subject**	**Estimate**	**Standard Error**	**Z Value**	**Pr Z**
**UN(1,1)**	Record_Id	16.6788	0.7360	22.66	<.0001
**UN(2,1)**	Record_Id	9.6582	0.6562	14.72	<.0001
**UN(2,2)**	Record_Id	16.7161	0.8517	19.63	<.0001
**UN(3,1)**	Record_Id	9.3249	0.7479	12.47	<.0001
**UN(3,2)**	Record_Id	9.6844	0.7776	12.45	<.0001
**UN(3,3)**	Record_Id	18.6519	1.0729	17.38	<.0001
**UN(4,1)**	Record_Id	8.6746	0.9703	8.94	<.0001
**UN(4,2)**	Record_Id	9.8265	1.0377	9.47	<.0001
**UN(4,3)**	Record_Id	12.4007	1.1426	10.85	<.0001
**UN(4,4)**	Record_Id	17.8570	1.5871	11.25	<.0001

Edinburgh Postnatal Depression Scale (EPDS)

The SAS System

The Mixed Procedure

Asymptotic Covariance Matrix of Estimates											
**Row**	**Cov Parm**	**CovP1**	**CovP2**	**CovP3**	**CovP4**	**CovP5**	**CovP6**	**CovP7**	**CovP8**	**CovP9**	**CovP10**
**1**	UN(1,1)	0.5417	0.3116	0.1776	0.3015	0.1719	0.1661	0.2786	0.1580	0.1524	0.1400
**2**	UN(2,1)	0.3116	0.4305	0.3958	0.2685	0.2963	0.2028	0.2687	0.2884	0.1996	0.1953
**3**	UN(2,2)	0.1776	0.3958	0.7254	0.2099	0.4113	0.2270	0.2191	0.4226	0.2361	0.2425
**4**	UN(3,1)	0.3015	0.2685	0.2099	0.5594	0.3791	0.5333	0.3687	0.2638	0.4119	0.3039
**5**	UN(3,2)	0.1719	0.2963	0.4113	0.3791	0.6047	0.5603	0.2755	0.4264	0.4609	0.3588
**6**	UN(3,3)	0.1661	0.2028	0.2270	0.5333	0.5603	1.1512	0.3274	0.3486	0.7546	0.4870
**7**	UN(4,1)	0.2786	0.2687	0.2191	0.3687	0.2755	0.3274	0.9416	0.6424	0.6735	0.9575
**8**	UN(4,2)	0.1580	0.2884	0.4226	0.2638	0.4264	0.3486	0.6424	1.0768	0.7421	1.1285
**9**	UN(4,3)	0.1524	0.1996	0.2361	0.4119	0.4609	0.7546	0.6735	0.7421	1.3055	1.4135
**10**	UN(4,4)	0.1400	0.1953	0.2425	0.3039	0.3588	0.4870	0.9575	1.1285	1.4135	2.5190

Fit Statistics	
**-2 Res Log Likelihood**	14216.2
**AIC (Smaller is Better)**	14236.2
**AICC (Smaller is Better)**	14236.2
**BIC (Smaller is Better)**	14285.6

Edinburgh Postnatal Depression Scale (EPDS)

The SAS System

The Mixed Procedure

Null Model Likelihood Ratio Test		
**DF**	**Chi-Square**	**Pr > ChiSq**
9	712.85	<.0001

Solution for Fixed Effects					
**Effect**	**Estimate**	**Standard Error**	**DF**	**t Value**	**Pr > |t|**
**Intercept**	16.9787	0.7568	1032	22.43	<.0001
**months**	-0.09284	0.03871	1032	-2.40	0.0167
**intervention**	0.2669	0.2650	1032	1.01	0.3141
**obese**	0.3990	0.4383	1032	0.91	0.3628
**W6LAS**	-0.1302	0.009290	1032	-14.01	<.0001
**months*intervention**	-0.06563	0.03128	1032	-2.10	0.0362
**months*obese**	0.2507	0.09972	1032	2.51	0.0121
**M6*monthsMinus6**	0.1757	0.05585	1032	3.15	0.0017
**obese*M6*monthsMinus**	-0.3917	0.1530	1032	-2.56	0.0106

Edinburgh Postnatal Depression Scale (EPDS)

The SAS System

The Mixed Procedure

Type 3 Tests of Fixed Effects				
**Effect**	**Num DF**	**Den DF**	**F Value**	**Pr > F**
**months**	1	1032	5.75	0.0167
**intervention**	1	1032	1.01	0.3141
**obese**	1	1032	0.83	0.3628
**W6LAS**	1	1032	196.36	<.0001
**months*intervention**	1	1032	4.40	0.0362
**months*obese**	1	1032	6.32	0.0121
**M6*monthsMinus6**	1	1032	9.89	0.0017
**obese*M6*monthsMinus**	1	1032	6.55	0.0106

Gotland Male Depression Scale (GMDS)

The SAS System

The Mixed Procedure

Model Information	
**Data Set**	WORK.M
**Dependent Variable**	MaleDepre
**Covariance Structure**	Unstructured
**Subject Effect**	Record_Id
**Estimation Method**	REML
**Residual Variance Method**	None
**Fixed Effects SE Method**	Model-Based
**Degrees of Freedom Method**	Between-Within

Gotland Male Depression Scale (GMDS)

The SAS System

The Mixed Procedure

Class Level Information		
**Class**	**Levels**	**Values**

Gotland Male Depression Scale (GMDS)

The SAS System

The Mixed Procedure

Class Level Information		
**Class**	**Levels**	**Values**
**Record_Id**	1044	AUG001 AUG002 AUG003 AUG004 AUG006 AUG007 AUG008 AUG009 AUG011 AUG012 AUG013 AUG014 AUG015 AUG016 AUG017 AUG019 AUG020 AUG021 AUG022 AUG024 AUG025 AUG026 AUG027 AUG028 AUG029 AUG030 AUG031 AUG033 AUG034 AUG035 AUG036 AUG037 AUG038 AUG039 AUG040 AUG041 AUG042 AUG043 AUG044 AUG045 AUG046 AUG047 AUG048 AUG049 AUG050 AUG051 AUG052 AUG053 AUG057 AUG058 AUG059 AUG061 AUG063 AUG064 AUG065 AUG066 AUG067 AUG068 AUG069 AUG070 AUG071 AUG080 AUG081 AUG083 AUG087 AUG089 AUG090 AUG095 AUG096 AUG097 AUG100 AUG101 AUG103 AUG104 AUG105 AUG107 AUG109 AUG110 AUG111 AUG113 AUG115 AUG116 AUG117 AUG127 AUG129 AUG130 AUG132 AUG135 AUG137 AUG138 AUG141 AUG143 AUG145 AUG147 AUG148 AUG150 AUG151 AUG152 AUG153 AUG155 AUG157 AUG158 AUG160 AUG161 AUG162 AUG164 AUG166 AUG169 AUG171 AUG172 AUG174 AUG176 AUG178 AUG179 AUG180 AUG181 AUG182 AUG184 AUG185 AUG186 AUG187 AUG188 AUG191 AUG193 AUG198 AUG201 AUG202 AUG203 AUG204 HEU001 HEU003 HEU004 HEU006 HEU007 HEU009 HEU010 HEU012 HEU013 HEU014 HEU015 HEU016 HEU017 HEU018 HEU019 HEU020 HEU021 HEU022 HEU023 HEU024 HEU025 HEU026 HEU027 HEU028 HEU029 HEU030 HEU031 HEU034 HEU035 HEU038 HEU040 HEU041 HEU042 HEU044 HEU045 HEU046 HEU047 HEU048 HEU049 HEU050 HEU051 HEU052 HEU054 HEU055 HEU056 HEU057 HEU058 HEU059 HEU060 HEU062 HEU063 HEU064 HEU065 HEU066 HEU067 HEU068 HEU069 HEU071 HEU072 HEU073 HEU074 HEU075 HEU076 HEU077 HEU078 HEU079 HEU081 HEU082 HEU083 HEU084 HEU085 HEU086 HEU087 HEU088 HEU089 HEU090 HEU091 HEU093 HEU095 HEU097 HEU098 HEU100 HEU102 HEU103 HEU104 HEU105 HEU106 HEU107 HEU108 HEU109 HEU110 HEU112 HEU113 HEU114 HEU115 HEU116 HEU117 HEU118 HEU119 HEU120 HEU121 HEU122 HEU123 HEU125 HEU126 HEU127 HEU128 HEU129 HEU130 HEU131 HEU133 HEU135 HEU136 HEU137 JES001 JES003 JES004 JES006 JES008 JES009 JES010 JES011 JES012 JES013 JES014 JES017 JES018 JES020 JES022 JES024 JES025 JES027 JES028 JES029 JES030 JES032 JES033 JES035 JES036 JES037 JES038 JES040 JES041 JES042 JES043 JES045 JES047 JES048 JES049 JES050 JES051 JES053 JES054 JES055 JES056 JES058 JES060 JES063 JES066 JES067 JES069 JES070 JES071 JES073 JES074 JES076 JES077 JES078 JES079 JES080 JES083 JES085 JES086 JES088

Gotland Male Depression Scale (GMDS)

The SAS System

The Mixed Procedure

Dimensions	
Covariance Parameters	10
Columns in X	3
Columns in Z	0
Subjects	1044
Max Obs per Subject	4

Number of Observations	
Number of Observations Read	2716
Number of Observations Used	1716
Number of Observations Not Used	1000

Iteration History

Iteration	Evaluations	-2 Res Log Like	Criterion
0	1	10369.68757124	
1	2	9968.24755501	0.00034006
2	1	9966.99312974	0.00000702
3	1	9966.96878005	0.00000001

Convergence criteria met.

Gotland Male Depression Scale (GMDS)

The SAS System

The Mixed Procedure

Covariance Parameter Estimates					
**Cov Parm**	**Subject**	**Estimate**	**Standard Error**	**Z Value**	**Pr Z**
**UN(1,1)**	Record_Id	18.0803	1.1381	15.89	<.0001
**UN(2,1)**	Record_Id	10.4682	1.0428	10.04	<.0001
**UN(2,2)**	Record_Id	22.2478	1.3889	16.02	<.0001
**UN(3,1)**	Record_Id	9.4313	1.2687	7.43	<.0001
**UN(3,2)**	Record_Id	14.7710	1.3762	10.73	<.0001
**UN(3,3)**	Record_Id	31.6410	1.9927	15.88	<.0001
**UN(4,1)**	Record_Id	9.6191	1.4861	6.47	<.0001
**UN(4,2)**	Record_Id	15.5035	1.8006	8.61	<.0001
**UN(4,3)**	Record_Id	18.6812	1.9549	9.56	<.0001
**UN(4,4)**	Record_Id	29.0542	2.6575	10.93	<.0001

Asymptotic Covariance Matrix of Estimates											
**Row**	**Cov Parm**	**CovP1**	**CovP2**	**CovP3**	**CovP4**	**CovP5**	**CovP6**	**CovP7**	**CovP8**	**CovP9**	**CovP10**
**1**	UN(1,1)	1.2953	0.6857	0.3034	0.5682	0.2333	0.1678	0.5510	0.2336	0.1723	0.1723
**2**	UN(2,1)	0.6857	1.0874	0.8651	0.6703	0.5896	0.3682	0.6423	0.5805	0.3613	0.3456
**3**	UN(2,2)	0.3034	0.8651	1.9291	0.4834	1.1479	0.6338	0.4729	1.1191	0.6103	0.5761
**4**	UN(3,1)	0.5682	0.6703	0.4834	1.6096	0.9392	1.1767	0.8958	0.5337	0.8638	0.5293
**5**	UN(3,2)	0.2333	0.5896	1.1479	0.9392	1.8939	1.7979	0.5522	1.0875	1.3441	0.8430
**6**	UN(3,3)	0.1678	0.3682	0.6338	1.1767	1.7979	3.9707	0.5558	0.8522	2.1512	1.0683

Gotland Male Depression Scale (GMDS)

The SAS System

The Mixed Procedure

Asymptotic Covariance Matrix of Estimates											
Row	Cov Parm	CovP1	CovP2	CovP3	CovP4	CovP5	CovP6	CovP7	CovP8	CovP9	CovP10
7	UN(4,1)	0.5510	0.6423	0.4729	0.8958	0.5522	0.5558	2.2085	1.4573	1.2467	1.8692
8	UN(4,2)	0.2336	0.5805	1.1191	0.5337	1.0875	0.8522	1.4573	3.2423	2.1842	3.3790
9	UN(4,3)	0.1723	0.3613	0.6103	0.8638	1.3441	2.1512	1.2467	2.1842	3.8216	3.6569
10	UN(4,4)	0.1723	0.3456	0.5761	0.5293	0.8430	1.0683	1.8692	3.3790	3.6569	7.0624

Fit Statistics	
-2 Res Log Likelihood	9967.0
AIC (Smaller is Better)	9987.0
AICC (Smaller is Better)	9987.1
BIC (Smaller is Better)	10036.5

Null Model Likelihood Ratio Test		
DF	Chi-Square	Pr > ChiSq
9	402.72	<.0001

Gotland Male Depression Scale (GMDS)

The SAS System

The Mixed Procedure

Solution for Fixed Effects					
**Effect**	**Estimate**	**Standard Error**	**DF**	**t Value**	**Pr > |t|**
**Intercept**	19.9808	0.9964	789	20.05	<.0001
**obese**	1.2080	0.4244	789	2.85	0.0045
**W6LAS**	-0.1802	0.01273	789	-14.16	<.0001

Type 3 Tests of Fixed Effects				
**Effect**	**Num DF**	**Den DF**	**F Value**	**Pr > F**
**obese**	1	789	8.10	0.0045
**W6LAS**	1	789	200.41	<.0001

Sense Of Coherence (SOC)

The SAS System

The Mixed Procedure

Model Information	
**Data Set**	WORK.B
**Dependent Variable**	SOC
**Covariance Structure**	Unstructured
**Subject Effect**	Record_Id
**Estimation Method**	REML
**Residual Variance Method**	None
**Fixed Effects SE Method**	Model-Based
**Degrees of Freedom Method**	Between-Within

Sense Of Coherence (SOC)

The SAS System

The Mixed Procedure

Class Level Information		
**Class**	**Levels**	**Values**

Sense Of Coherence (SOC)

The SAS System

The Mixed Procedure

Class Level Information		
**Class**	**Levels**	**Values**
**Record_Id**	1044	AUG001 AUG002 AUG003 AUG004 AUG006<br>AUG007 AUG008 AUG009 AUG011 AUG012<br>AUG013 AUG014 AUG015 AUG016 AUG017<br>AUG019 AUG020 AUG021 AUG022 AUG024<br>AUG025 AUG026 AUG027 AUG028 AUG029<br>AUG030 AUG031 AUG033 AUG034 AUG035<br>AUG036 AUG037 AUG038 AUG039 AUG040<br>AUG041 AUG042 AUG043 AUG044 AUG045<br>AUG046 AUG047 AUG048 AUG049 AUG050<br>AUG051 AUG052 AUG053 AUG057 AUG058<br>AUG059 AUG061 AUG063 AUG064 AUG065<br>AUG066 AUG067 AUG068 AUG069 AUG070<br>AUG071 AUG080 AUG081 AUG083 AUG087<br>AUG089 AUG090 AUG095 AUG096 AUG097<br>AUG100 AUG101 AUG103 AUG104 AUG105<br>AUG107 AUG109 AUG110 AUG111 AUG113<br>AUG115 AUG116 AUG117 AUG127 AUG129<br>AUG130 AUG132 AUG135 AUG137 AUG138<br>AUG141 AUG143 AUG145 AUG147 AUG148<br>AUG150 AUG151 AUG152 AUG153 AUG155<br>AUG157 AUG158 AUG160 AUG161 AUG162<br>AUG164 AUG166 AUG169 AUG171 AUG172<br>AUG174 AUG176 AUG178 AUG179 AUG180<br>AUG181 AUG182 AUG184 AUG185 AUG186<br>AUG187 AUG188 AUG191 AUG193 AUG198<br>AUG201 AUG202 AUG203 AUG204 HEU001<br>HEU003 HEU004 HEU006 HEU007 HEU009<br>HEU010 HEU012 HEU013 HEU014 HEU015<br>HEU016 HEU017 HEU018 HEU019 HEU020<br>HEU021 HEU022 HEU023 HEU024 HEU025<br>HEU026 HEU027 HEU028 HEU029 HEU030<br>HEU031 HEU034 HEU035 HEU038 HEU040<br>HEU041 HEU042 HEU044 HEU045 HEU046<br>HEU047 HEU048 HEU049 HEU050 HEU051<br>HEU052 HEU054 HEU055 HEU056 HEU057<br>HEU058 HEU059 HEU060 HEU062 HEU063<br>HEU064 HEU065 HEU066 HEU067 HEU068<br>HEU069 HEU071 HEU072 HEU073 HEU074<br>HEU075 HEU076 HEU077 HEU078 HEU079<br>HEU081 HEU082 HEU083 HEU084 HEU085<br>HEU086 HEU087 HEU088 HEU089 HEU090<br>HEU091 HEU093 HEU095 HEU097 HEU098<br>HEU100 HEU102 HEU103 HEU104 HEU105<br>HEU106 HEU107 HEU108 HEU109 HEU110<br>HEU112 HEU113 HEU114 HEU115 HEU116<br>HEU117 HEU118 HEU119 HEU120 HEU121<br>HEU122 HEU123 HEU125 HEU126 HEU127<br>HEU128 HEU129 HEU130 HEU131 HEU133<br>HEU135 HEU136 HEU137 JES001 JES003 JES004<br>JES006 JES008 JES009 JES010 JES011 JES012<br>JES013 JES014 JES017 JES018 JES020 JES022<br>JES024 JES025 JES027 JES028 JES029 JES030<br>JES032 JES033 JES035 JES036 JES037 JES038<br>JES040 JES041 JES042 JES043 JES045 JES047<br>JES048 JES049 JES050 JES051 JES053 JES054<br>JES055 JES056 JES058 JES060 JES063 JES066<br>JES067 JES069 JES070 JES071 JES073 JES074<br>JES076 JES077 JES078 JES079 JES080 JES083<br>JES085 JES086 JES088 JES091 JES092 JES093

Sense Of Coherence (SOC)

The SAS System

The Mixed Procedure

Dimensions	
Covariance Parameters	10
Columns in X	16
Columns in Z	0
Subjects	1044
Max Obs per Subject	4

Number of Observations	
Number of Observations Read	2716
Number of Observations Used	2672
Number of Observations Not Used	44

Iteration History			
Iteration	Evaluations	-2 Res Log Like	Criterion
0	1	20278.97542729	
1	2	19071.74292831	0.00007769
2	1	19071.16712254	0.00000040
3	1	19071.16425636	0.00000000

Convergence criteria met.

Estimated R Matrix for Record_Id AUG001	
Row	Col1
1	101.80

Covariance Parameter Estimates					
Cov Parm	Subject	Estimate	Standard Error	Z Value	Pr Z
UN(1,1)	Record_Id	101.80	4.4745	22.75	<.0001
UN(2,1)	Record_Id	79.3198	4.5361	17.49	<.0001
UN(2,2)	Record_Id	121.96	6.0963	20.01	<.0001

Sense Of Coherence (SOC)

The SAS System

The Mixed Procedure

Covariance Parameter Estimates					
**Cov Parm**	**Subject**	**Estimate**	**Standard Error**	**Z Value**	**Pr Z**
**UN(3,1)**	Record_Id	73.9932	4.9382	14.98	<.0001
**UN(3,2)**	Record_Id	89.0026	5.7435	15.50	<.0001
**UN(3,3)**	Record_Id	131.80	7.4135	17.78	<.0001
**UN(4,1)**	Record_Id	81.0126	6.4875	12.49	<.0001
**UN(4,2)**	Record_Id	92.5339	7.4216	12.47	<.0001
**UN(4,3)**	Record_Id	107.82	8.2212	13.11	<.0001
**UN(4,4)**	Record_Id	150.03	11.8944	12.61	<.0001

Asymptotic Covariance Matrix of Estimates											
**Row**	**Cov Parm**	**CovP1**	**CovP2**	**CovP3**	**CovP4**	**CovP5**	**CovP6**	**CovP7**	**CovP8**	**CovP9**	**CovP10**
**1**	UN(1,1)	20.0210	15.5739	12.1046	14.5347	11.2958	10.5351	15.9016	12.3583	11.5289	12.6162
**2**	UN(2,1)	15.5739	20.5765	22.5663	15.5912	18.2035	14.4543	16.3997	19.4525	15.3366	16.2555
**3**	UN(2,2)	12.1046	22.5663	37.1644	15.4647	26.3724	18.5688	15.9049	27.3575	19.1980	19.8351
**4**	UN(3,1)	14.5347	15.5912	15.4647	24.3859	22.1130	27.8194	20.0106	18.9955	25.5652	22.6490
**5**	UN(3,2)	11.2958	18.2035	26.3724	22.1130	32.9883	34.0717	18.5311	27.7810	30.6241	26.8341
**6**	UN(3,3)	10.5351	14.4543	18.5688	27.8194	34.0717	54.9595	20.7412	25.9249	43.6740	34.1627
**7**	UN(4,1)	15.9016	16.3997	15.9049	20.0106	18.5311	20.7412	42.0881	35.3237	37.2193	56.5260
**8**	UN(4,2)	12.3583	19.4525	27.3575	18.9955	27.7810	25.9249	35.3237	55.0797	46.3711	67.2641
**9**	UN(4,3)	11.5289	15.3366	19.1980	25.5652	30.6241	43.6740	37.2193	46.3711	67.5887	81.0460
**10**	UN(4,4)	12.6162	16.2555	19.8351	22.6490	26.8341	34.1627	56.5260	67.2641	81.0460	141.48

Fit Statistics	
**-2 Res Log Likelihood**	19071.2
**AIC (Smaller is Better)**	19091.2
**AICC (Smaller is Better)**	19091.2
**BIC (Smaller is Better)**	19140.7

Null Model Likelihood Ratio Test		
**DF**	**Chi-Square**	**Pr > ChiSq**
9	1207.81	<.0001

Sense Of Coherence (SOC)

The SAS System

The Mixed Procedure

Solution for Fixed Effects					
**Effect**	**Estimate**	**Standard Error**	**DF**	**t Value**	**Pr > |t|**
**Intercept**	34.2899	2.0697	1036	16.57	<.0001
**months**	0.2184	0.1257	1036	1.74	0.0826
**intervention**	3.0717	1.0092	1036	3.04	0.0024
**overweight**	3.2068	1.1245	1036	2.85	0.0044
**obese**	0.2242	1.2905	1036	0.17	0.8621
**W6LAS**	0.4410	0.02430	1036	18.15	<.0001
**exc**	-0.1607	0.07779	1036	-2.07	0.0391
**intervent*overweight**	-5.5089	1.5787	1036	-3.49	0.0005
**intervention*obese**	-5.0964	1.7503	1036	-2.91	0.0037
**months*intervention**	-0.2988	0.1882	1036	-1.59	0.1127
**months*overweight**	-0.6112	0.2161	1036	-2.83	0.0048
**months*interv*overwe**	1.0320	0.3159	1036	3.27	0.0011
**M6*monthsMinus6**	-0.2100	0.1972	1036	-1.06	0.2873
**interven*M6*monthsMi**	0.2577	0.2861	1036	0.90	0.3679
**overweig*M6*monthsMi**	0.6967	0.3433	1036	2.03	0.0427
**inter*overw*M6*month**	-1.0755	0.4851	1036	-2.22	0.0268

Type 3 Tests of Fixed Effects				
**Effect**	**Num DF**	**Den DF**	**F Value**	**Pr > F**
**months**	1	1036	3.02	0.0826
**intervention**	1	1036	9.26	0.0024
**overweight**	1	1036	8.13	0.0044
**obese**	1	1036	0.03	0.8621
**W6LAS**	1	1036	329.42	<.0001
**exc**	1	1036	4.27	0.0391
**intervent*overweight**	1	1036	12.18	0.0005
**intervention*obese**	1	1036	8.48	0.0037
**months*intervention**	1	1036	2.52	0.1127
**months*overweight**	1	1036	8.00	0.0048
**months*interv*overwe**	1	1036	10.67	0.0011
**M6*monthsMinus6**	1	1036	1.13	0.2873
**interven*M6*monthsMi**	1	1036	0.81	0.3679

Sense Of Coherence (SOC)

The SAS System

The Mixed Procedure

Type 3 Tests of Fixed Effects				
**Effect**	**Num DF**	**Den DF**	**F Value**	**Pr > F**
**overweig*M6*monthsMi**	1	1036	4.12	0.0427
**inter*overw*M6*month**	1	1036	4.92	0.0268

Quality of Life (QoL)

The SAS System

The Mixed Procedure

Model Information	
**Data Set**	WORK.B
**Dependent Variable**	LAS
**Covariance Structure**	Unstructured
**Subject Effect**	Record_Id
**Estimation Method**	REML
**Residual Variance Method**	None
**Fixed Effects SE Method**	Model-Based
**Degrees of Freedom Method**	Between-Within

Quality of Life (QoL)

The SAS System

The Mixed Procedure

Class Level Information		
**Class**	**Levels**	**Values**

Quality of Life (QoL)

The SAS System

The Mixed Procedure

Class Level Information		
**Class**	**Levels**	**Values**
**Record_Id**	1039	AUG001 AUG002 AUG003 AUG004 AUG006 AUG007 AUG008 AUG009 AUG011 AUG012 AUG013 AUG014 AUG015 AUG016 AUG017 AUG019 AUG020 AUG021 AUG022 AUG024 AUG025 AUG026 AUG027 AUG028 AUG029 AUG030 AUG031 AUG033 AUG034 AUG035 AUG036 AUG037 AUG038 AUG039 AUG040 AUG041 AUG042 AUG043 AUG044 AUG045 AUG046 AUG047 AUG048 AUG049 AUG050 AUG051 AUG052 AUG053 AUG057 AUG058 AUG059 AUG061 AUG063 AUG064 AUG065 AUG066 AUG067 AUG068 AUG069 AUG070 AUG071 AUG080 AUG081 AUG083 AUG087 AUG089 AUG090 AUG095 AUG096 AUG097 AUG100 AUG101 AUG103 AUG104 AUG105 AUG107 AUG109 AUG110 AUG111 AUG113 AUG115 AUG116 AUG117 AUG127 AUG129 AUG130 AUG132 AUG135 AUG137 AUG138 AUG141 AUG143 AUG145 AUG147 AUG148 AUG150 AUG151 AUG152 AUG153 AUG155 AUG157 AUG158 AUG160 AUG161 AUG162 AUG164 AUG166 AUG169 AUG171 AUG172 AUG174 AUG176 AUG178 AUG179 AUG180 AUG181 AUG182 AUG184 AUG185 AUG186 AUG187 AUG188 AUG191 AUG193 AUG198 AUG201 AUG202 AUG203 AUG204 HEU001 HEU003 HEU004 HEU006 HEU007 HEU009 HEU010 HEU012 HEU013 HEU014 HEU015 HEU016 HEU017 HEU018 HEU019 HEU020 HEU021 HEU022 HEU023 HEU024 HEU025 HEU026 HEU027 HEU028 HEU029 HEU030 HEU031 HEU034 HEU035 HEU038 HEU040 HEU041 HEU042 HEU044 HEU045 HEU046 HEU047 HEU048 HEU049 HEU050 HEU051 HEU052 HEU054 HEU055 HEU056 HEU057 HEU058 HEU059 HEU060 HEU062 HEU063 HEU064 HEU065 HEU066 HEU067 HEU068 HEU069 HEU071 HEU072 HEU073 HEU074 HEU075 HEU076 HEU077 HEU078 HEU079 HEU081 HEU082 HEU083 HEU084 HEU085 HEU086 HEU087 HEU088 HEU089 HEU090 HEU091 HEU093 HEU095 HEU097 HEU098 HEU100 HEU102 HEU103 HEU104 HEU105 HEU106 HEU107 HEU108 HEU109 HEU110 HEU112 HEU113 HEU114 HEU115 HEU116 HEU117 HEU118 HEU119 HEU120 HEU121 HEU122 HEU123 HEU125 HEU126 HEU127 HEU128 HEU129 HEU130 HEU131 HEU133 HEU135 HEU136 HEU137 JES001 JES003 JES004 JES006 JES008 JES009 JES010 JES011 JES012 JES013 JES014 JES017 JES018 JES020 JES022 JES024 JES025 JES027 JES028 JES029 JES030 JES032 JES033 JES035 JES036 JES037 JES038 JES040 JES041 JES042 JES043 JES045 JES047 JES048 JES049 JES050 JES051 JES053 JES054 JES055 JES056 JES058 JES060 JES063 JES066 JES067 JES069 JES070 JES071 JES073 JES074 JES076 JES077 JES078 JES079 JES080 JES083 JES085 JES086 JES088

Quality of Life (QoL)

The SAS System

The Mixed Procedure

Dimensions	
**Covariance Parameters**	10
**Columns in X**	10
**Columns in Z**	0
**Subjects**	1039
**Max Obs per Subject**	4

Number of Observations	
**Number of Observations Read**	2622
**Number of Observations Used**	2622
**Number of Observations Not Used**	0

Iteration History			
**Iteration**	**Evaluations**	**-2 Res Log Like**	**Criterion**
**0**	1	18469.13997333	
**1**	2	17287.00438433	0.00617271
**2**	1	17241.40193333	0.00087573
**3**	1	17235.28174264	0.00005739
**4**	1	17234.89460175	0.00000141
**5**	1	17234.88563860	0.00000000

Quality of Life (QoL)

The SAS System

The Mixed Procedure

Convergence criteria met.

Estimated R Matrix for Record_Id AUG001	
**Row**	**Col1**
**1**	11.7844

Covariance Parameter Estimates					
**Cov Parm**	**Subject**	**Estimate**	**Standard Error**	**Z Value**	**Pr Z**
**UN(1,1)**	Record_Id	11.7844	0.5502	21.42	<.0001
**UN(2,1)**	Record_Id	-1.6932	1.7953	-0.94	0.3456
**UN(2,2)**	Record_Id	110.95	5.8295	19.03	<.0001
**UN(3,1)**	Record_Id	-12.1837	2.5747	-4.73	<.0001
**UN(3,2)**	Record_Id	51.1692	5.4676	9.36	<.0001
**UN(3,3)**	Record_Id	122.14	8.1927	14.91	<.0001
**UN(4,1)**	Record_Id	-21.7113	4.5910	-4.73	<.0001
**UN(4,2)**	Record_Id	49.6444	8.3309	5.96	<.0001
**UN(4,3)**	Record_Id	77.3987	9.6340	8.03	<.0001
**UN(4,4)**	Record_Id	143.09	17.5733	8.14	<.0001

Quality of Life (QoL)

The SAS System

The Mixed Procedure

Asymptotic Covariance Matrix of Estimates											
**Row**	**Cov Parm**	**CovP1**	**CovP2**	**CovP3**	**CovP4**	**CovP5**	**CovP6**	**CovP7**	**CovP8**	**CovP9**	**CovP10**
**1**	UN(1,1)	0.3027	-0.04114	-0.2835	-0.2731	-0.2186	-0.07234	-0.4428	-0.1084	0.2045	0.4905
**2**	UN(2,1)	-0.04114	3.2232	-0.8913	1.6147	-3.3850	-3.1387	1.0534	-6.0196	-3.9084	-3.7134
**3**	UN(2,2)	-0.2835	-0.8913	33.9833	-0.5166	17.0617	10.1174	-0.7548	16.3703	9.6011	10.2292
**4**	UN(3,1)	-0.2731	1.6147	-0.5166	6.6290	-0.8803	-11.8479	3.3109	-2.7792	-14.1465	-10.8067
**5**	UN(3,2)	-0.2186	-3.3850	17.0617	-0.8803	29.8947	23.4279	-1.4855	20.8397	18.7038	15.8264
**6**	UN(3,3)	-0.07234	-3.1387	10.1174	-11.8479	23.4279	67.1197	-6.3917	16.5399	50.3250	35.0876
**7**	UN(4,1)	-0.4428	1.0534	-0.7548	3.3109	-1.4855	-6.3917	21.0771	4.4069	-18.8951	-65.2828
**8**	UN(4,2)	-0.1084	-6.0196	16.3703	-2.7792	20.8397	16.5399	4.4069	69.4031	34.4618	33.8728
**9**	UN(4,3)	0.2045	-3.9084	9.6011	-14.1465	18.7038	50.3250	-18.8951	34.4618	92.8143	111.69
**10**	UN(4,4)	0.4905	-3.7134	10.2292	-10.8067	15.8264	35.0876	-65.2828	33.8728	111.69	308.82

Fit Statistics	
**-2 Res Log Likelihood**	17234.9
**AIC (Smaller is Better)**	17254.9
**AICC (Smaller is Better)**	17255.0
**BIC (Smaller is Better)**	17304.3

Quality of Life (QoL)

The SAS System

The Mixed Procedure

Null Model Likelihood Ratio Test		
DF	Chi-Square	Pr > ChiSq
9	1234.25	<.0001

Solution for Fixed Effects					
Effect	Estimate	Standard Error	DF	t Value	Pr > |t|
Intercept	10.5651	0.7015	1035	15.06	<.0001
months	0.9905	0.1287	1035	7.70	<.0001
overweight	1.4762	0.4814	1035	3.07	0.0022
obese	2.0396	0.6577	1035	3.10	0.0020
W6LAS	0.8417	0.007755	1035	108.52	<.0001
months*overweight	-0.8943	0.2053	1035	-4.36	<.0001
months*obese	-1.4375	0.2839	1035	-5.06	<.0001
M6*monthsMinus6	-1.5487	0.1947	1035	-7.95	<.0001
overweig*M6*monthsMi	1.3980	0.3124	1035	4.47	<.0001
obese*M6*monthsMinus	2.5052	0.4330	1035	5.79	<.0001

Quality of Life (QoL)

The SAS System

The Mixed Procedure

Type 3 Tests of Fixed Effects				
**Effect**	**Num DF**	**Den DF**	**F Value**	**Pr > F**
**months**	1	1035	59.22	<.0001
**overweight**	1	1035	9.40	0.0022
**obese**	1	1035	9.62	0.0020
**W6LAS**	1	1035	11777.4	<.0001
**months*overweight**	1	1035	18.97	<.0001
**months*obese**	1	1035	25.64	<.0001
**M6*monthsMinus6**	1	1035	63.25	<.0001
**overweig*M6*monthsMi**	1	1035	20.02	<.0001
**obese*M6*monthsMinus**	1	1035	33.47	<.0001
